# “Smart” drug delivery: A window to future of translational medicine

**DOI:** 10.3389/fchem.2022.1095598

**Published:** 2023-01-04

**Authors:** Abhilash Rana, Meheli Adhikary, Praveen Kumar Singh, Bhudev C. Das, Seema Bhatnagar

**Affiliations:** ^1^ Amity Institute of Biotechnology, Amity University, Noida, Uttar Pradesh, India; ^2^ Amity Institute of Molecular Medicine and Stem Cell Research, Amity University, Noida, Uttar Pradesh, India

**Keywords:** smart drug delivery systems (SDDSs), cancer, translational medicine, nano-therapy, active targeting, passive targeting, targeted drug delivery, targeted cancer therapy

## Abstract

Chemotherapy is the mainstay of cancer treatment today. Chemotherapeutic drugs are non-selective and can harm both cancer and healthy cells, causing a variety of adverse effects such as lack of specificity, cytotoxicity, short half-life, poor solubility, multidrug resistance, and acquiring cancer stem-like characteristics. There is a paradigm shift in drug delivery systems (DDS) with the advent of smarter ways of targeted cancer treatment. Smart Drug Delivery Systems (SDDSs) are stimuli responsive and can be modified in chemical structure in response to light, pH, redox, magnetic fields, and enzyme degradation can be future of translational medicine. Therefore, SDDSs have the potential to be used as a viable cancer treatment alternative to traditional chemotherapy. This review focuses mostly on stimuli responsive drug delivery, inorganic nanocarriers (Carbon nanotubes, gold nanoparticles, Meso-porous silica nanoparticles, quantum dots *etc.*), organic nanocarriers (Dendrimers, liposomes, micelles), antibody-drug conjugates (ADC) and small molecule drug conjugates (SMDC) based SDDSs for targeted cancer therapy and strategies of targeted drug delivery systems in cancer cells.

## Graphical Abstract

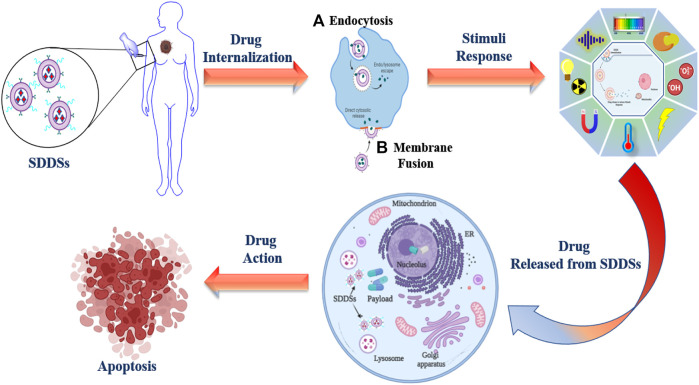

## 1 Introduction

Cancer has emerged as a leading health concern of the 21st century, with over 10 million new patients diagnosed each year (Sung et al., 2021). In 2020, more than 19 million people worldwide were diagnosed with cancer, with nearly 10 million dying as a result (Mao et al., 2022). By 2040, the number of new cases and deaths is expected to be around 28 million and 16 million, respectively (Khazaei et al., 2021; Sung et al., 2021). Currently, surgical resection, radiation therapy (RT), and chemotherapy are the three major treatment modalities of cancer treatment. The comparative usefulness of various procedures are determined on the basis and type of cancer and stage of development. Despite recent advances in treatment strategies and targeted treatment, the survival rate has not improved significantly. As a result, innovative cancer treatment approaches are required. Chemotherapy has been one of the most effective treatments for both localised and metastatic tumours for more than 50 years. The issue of systemic side effects from chemotherapy has yet to be addressed. Conventional drug delivery systems frequently have systemic adverse effects due to non-specific biological distribution and uncontrolled drug release features. Exploration of innovative drug delivery technology can have commercial as well as therapeutic value for health products, is needed to have significant expansion (Liu et al., 2016). Moreover, many drugs are difficult to administer using traditional drug delivery techniques due to a lack of therapeutic effectiveness and a variety of challenges such as limited bioavailability, sensitive toxicity, insufficient specificity, and so on (Majumder and Minko, 2021). Additionally, challenges to be consider and overcome, include the attack of enzymes, the poor permeability of some tissues, and the difficulty of access to the target once arriving at the destination cells, among others (Alvarez-Lorenzo and Concheiro, 2014). There is a need to investigate new innovative ways of drug delivery that can minimize side effects. A drug delivery system (DDS) is a method or process that releases the drug at a pre-selected site in a controlled manner to achieve therapeutic effect. Drug delivery systems can in principle provide enhanced efficacy and/or reduced toxicity for a therapeutic agents. An ideal DDS in cancer achieves two goals: tumor-specific delivery and tumor-specific drug release from delivery systems (Nkepang et al., 2014). Smart Drug Delivery Systems (SDDSs) were developed to circumvent these limitations, allowing payloads to be delivered to target areas in a spatially controlled way. SDDS have many other applications and can be developed into smart systems, encasing therapeutic and imaging agents as well as bearing stealth property. SDDSs can also be used to develop diagnostics tools, PET scanning, MRI-CAs for efficient and early diagnosis of cancer (Wu and Wang, 2016).

Targeted treatments aim to block specific biologic transduction pathways or cancer proteins that are involved in tumour growth and progression, i.e., molecular targets (receptors, kinase cascades, growth factors, or molecules related to angiogenesis and apoptosis) that are found overexpressed or mutated in cancer (Chabner et al., 2005; Hanahan and Weinberg, 2011). The primary objective of these revolutionary therapies is to either block the signals that lead cancer cells to grow and divide uncontrollably, induce apoptosis in cancer cells, stimulate the immune system, or target the delivery of chemotherapy agents specifically to cancer cells, minimising the death of normal cells and avoiding the negative side effects (Perez and Fernandez-Medarde, 2015).

SDDSs can preferentially accumulate and bind to the disease target, allowing for controlled release. It is common knowledge that drugs should be released at target areas in a regulated way to maximise therapeutic effectiveness while minimising negative effects. The loaded medicines can act “smart” by inheriting from the controlled release (Liu et al., 2016).

SDDSs are designed to take advantage of the different conditions (e.g., temperature, pH, and enzyme concentration) that occur in pathological tissues rather than in normal tissues in a “smart” way, enabling them to trigger drug release in the targeted tissue, overcome intermediate barriers, and increase bioavailability, blood circulation time, and overall therapeutic efficacy (AlSawaftah et al., 2021). A better understanding of tumour biology, combined with the increased availability of versatile materials such as polymers, lipids, inorganic carriers, polymeric hydrogels, and bio-macromolecular scaffolds, has led to the development of systems that can deliver chemotherapeutics to tumour sites with improved therapeutic efficacy in recent years (Senapati et al., 2018).

Drug delivery efficiency refers to the safe delivery of a drug to target locations without significant off-target effects (Sanadgol and Wackerlig, 2020). SDDSs are efficient tools to ensure the release of the therapeutic agent at the target and in the right dosage for the needed duration in order to optimise its efficacy by accumulating at the site of action and achieve the therapeutic effective concentration level within the therapeutic window while minimising adverse effects on healthy tissues. This delivery method must be biocompatible and biodegradable in order to penetrate the tissue and cells without causing specific toxicity, immunogenicity, or accumulation in organs other than the tumour. SDDSs have the potential to deliver medicines to precise and targeted locations. The most reported carriers mainly are Liposomes, micelles, dendrimers, meso-porous silica nanoparticles (MSNs), and gold nanoparticles, carbon nanotubes (CNTs), quantum dots (QDs), vitamins (Folic acid (B9) (Rana and Bhatnagar, 2021) and Biotin (B7) (Saha et al., 2013) and monoclonal antibodies (Kimiz-Gebologlu et al., 2018).

In this review article, we highlight the recent development of various SDDSs used in cancer therapeutics to increase the therapeutic index of chemotherapeutic drugs. We highlighted the components and classification of SDDSs, example of target nanocarriers, antibody based smart drug delivery systems, small molecule based smart drug delivery systems. In the context of the current oncological developments, the contribution of fundamental research to clinical practices with respect to SDDSs is explored.

## 2 smart drug delivery systems (SDDSs)

SDDSs have the exciting potential to vastly improve the efficiency and precision of treatment across a wide range of disorders. Smart drug delivery is a means of administering treatment to a patient in a targeted and controlled release manner. SDDSs can efficiently lower dosage frequency while maintaining drug concentrations in certain organs or tissues for a longer period of time when compared to conventional DDSs. In this way, SDDSs offer a wealth of possibilities for lowering drug concentration fluctuations, reducing drug toxicity, and enhancing therapeutic efficacy.

Most anticancer drugs are given at the maximum tolerated dose, cancer patients frequently suffer from severe cytotoxic side effects, limiting their treatment options. SDDSs allow for lower drug doses while maintaining effective intracellular concentrations, therefore expanding the therapeutic window of anticancer drugs. SDDSs have several advantages, including improved specific localization, patient compliance, reduced toxic side effects, and controlled biodistribution (Naziris et al., 2016).

### 2.1 Components of SDDS

**FIGURE 1:**
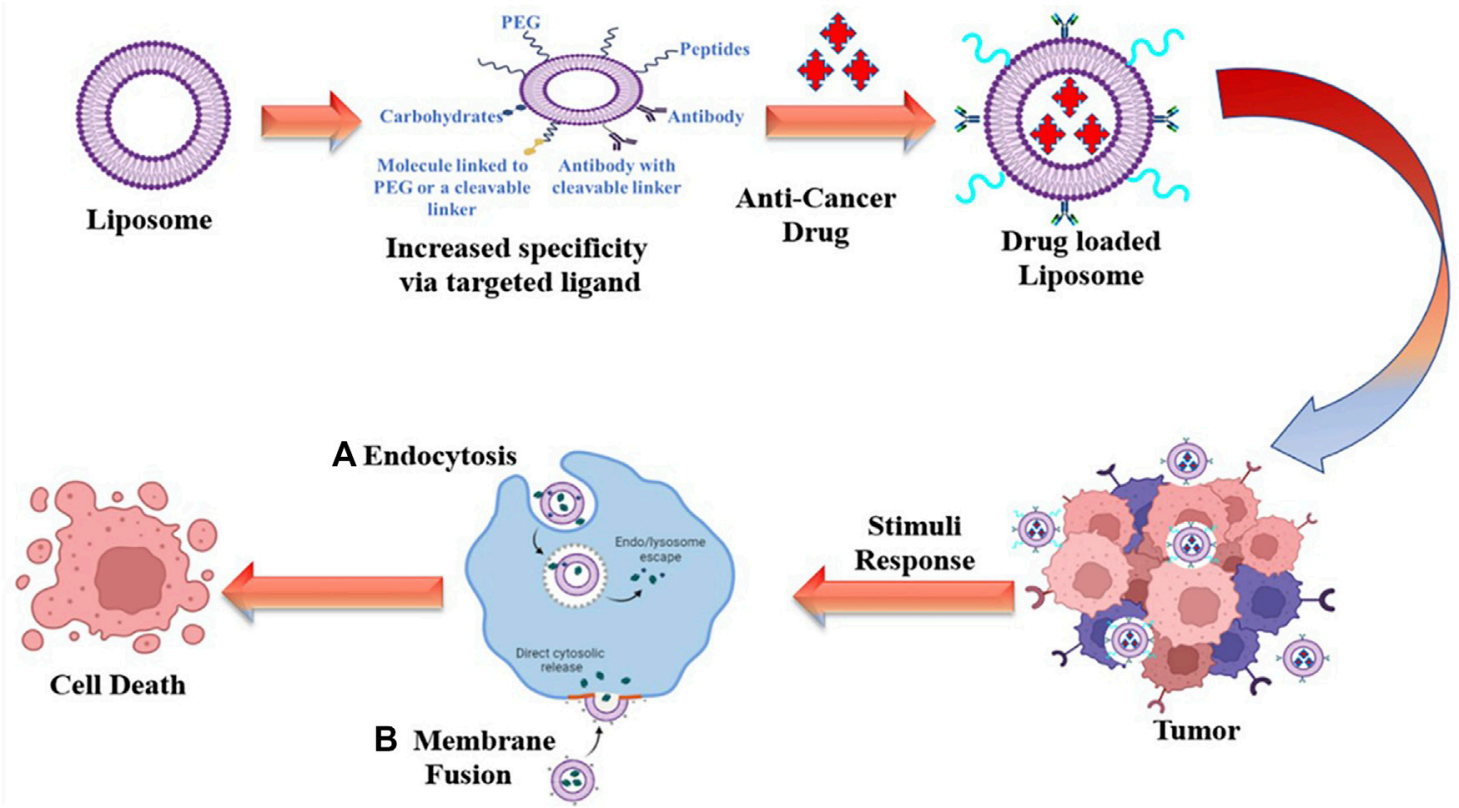
Overview of a SDDSs using liposomes as smart carrier.

Successful drug delivery requires that drugs should be released at desired target sites in a controlled manner to maximise therapeutic efficacy while minimising side effects. SDDSs, are made up of the following components: carriers/targeting ligand, linker and cytotoxic drug payload. A smart drug delivery system (SDDS) using liposomes as smart carrier (Figure 1), consists of (Liu et al.) Smart Carriers/Targeting Ligands that transport anti-cancer drugs to the targeted cancer site, (ii) targeting mechanisms that locate the cancerous site, and (iii) stimulus techniques that release the payload drugs at the pre-located cancer cell site. The following sections go over the various SDDSs, as well as their targeting mechanisms and stimulus techniques.

#### 2.1.1 Targets utilized by SDDS

The drug target is a crucial part of SDDSs. Commonly explored drug targets in the body: **i)** Receptors on cell membranes which enable drug carriers to engage specifically with cells, boosting drug absorption *via* receptor-mediated endocytosis. For example, folate receptors (FRs), which are differentially overexpressed in epithelial cancer cells, are used to deliver tumor-specific drugs in cancers such as breast, ovarian, brain, and lung cancers (Rana and Bhatnagar, 2021). G protein coupled receptors (GPCRs), Integrins, sigma receptors, Epidermal growth factor receptor, Sigma receptors (SRs) these over expressed receptors are frequently used in preclinical cancer models for selective drug delivery *via* receptor–ligand pairs (Rana and Bhatnagar, 2021). Follicle-stimulating hormone receptors, C-type lectin receptors, biotin receptors, and neuropilin receptors are some of the less common receptors that have lately been utilised (Kim et al., 2018). Other targets for tumor-selective accumulation of drug carriers include those expressed on tumour vasculature endothelial cells. The dependence of tumour growth on angiogenesis is a potential target for the development of therapies to prevent the production of new tumor-feeding blood vessels to reduce tumour progression (Xu J. et al., 2017). Anti-angiogenesis strategies are successful in reducing tumour development, with endothelial cells in tumour blood arteries being the primary targets. Cancer cells are denied of nutrition and oxygen, resulting in the tumor death (Teleanu et al., 2019). Targeting ligands are coupled to drug-loaded nanocarriers. These ligands find their corresponding target on the cancer cell surface, which is overexpressed. A wide variety of synthetic and natural chemicals of various chemical classes have been utilised to target nano systems against cancer cells. Antibodies (Ab) and other proteins (such as transferrin), Aptamers, tiny molecules like folic acid, and peptides are among the most utilised. It is important to identify optimum targets to maximise the efficacy of active targeting. Identifying receptors expressed at greater levels on target cancer cells than on normal cells is the justification for picking optimal targets (Gui et al., 2017).

ii) The Cell Membrane Lipid Components: When synthetic phospholipid analogues interact with biological membranes, they change the lipid content, membrane permeability, and fluidity. As a result, signal transduction pathways are disrupted, resulting in apoptosis (Torres et al., 2021). iii) Cell Surface Antigens or Proteins: The diseased cells either produce novel proteins or show differential (under/over) expression of proteins seen in healthy cells. Against such proteins, monoclonal antibodies are employed. The tumor-specific antigen that may be used to target drugs is one that is expressed exclusively and uniformly by all tumour cells (Khanna, 2012).

#### 2.1.2 Targeting ligands

**FIGURE 2:**
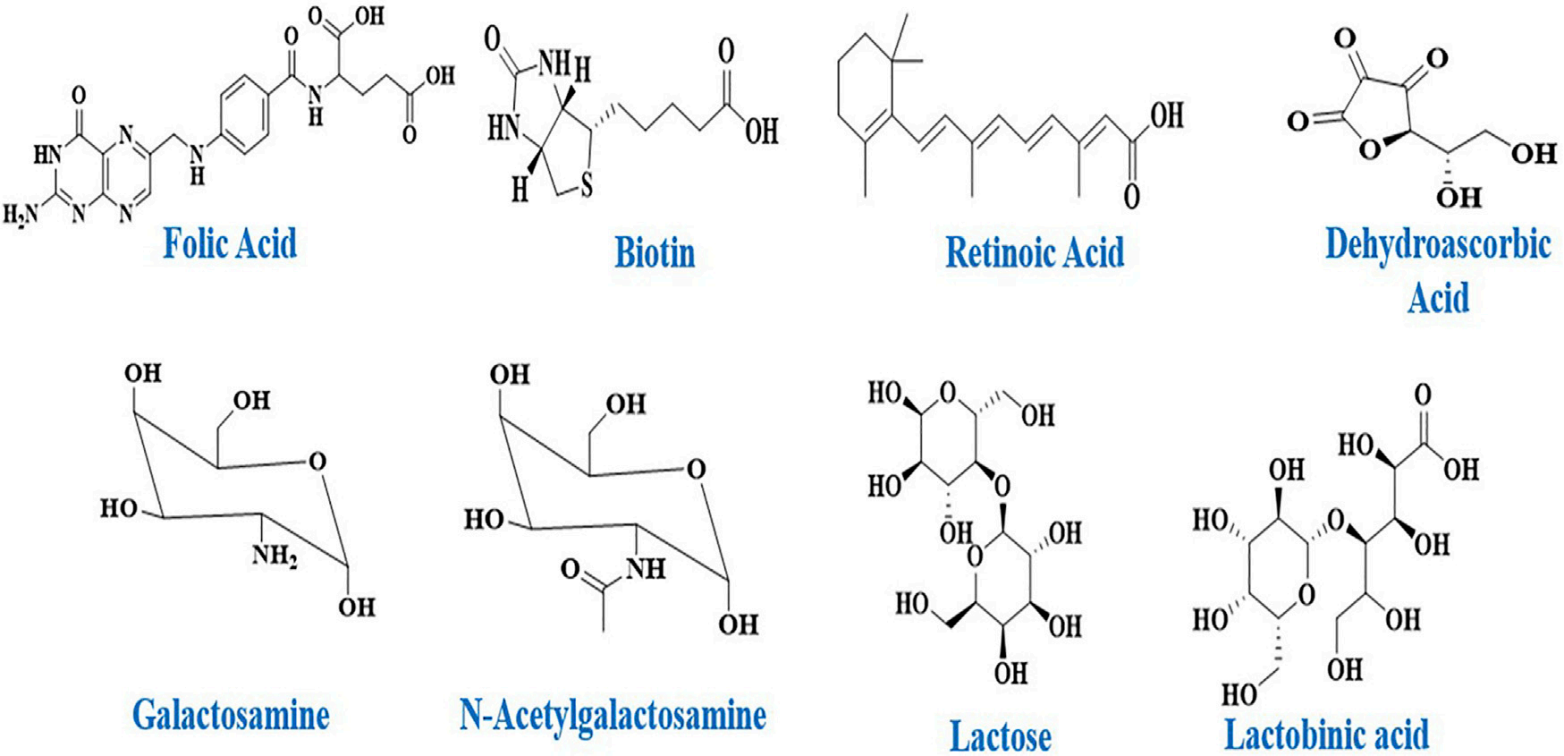
Some example of Targeting Ligands utilized in SDDSs.

Sugars, folic acid, peptides, monoclonal-antibodies and specifically designed antibodies are examples of ligands that bind with specific receptors found on certain cell types with some degree of exclusivity. Nucleic acids like aptamers, tiny compounds like vitamins, and sugars like galactose, mannose, and other sugars have also been described as cellular targeting components (Figure 2) (Khanna, 2012).

#### 2.1.3 Carrier and Targeting ligand (TL)

**FIGURE 3:**
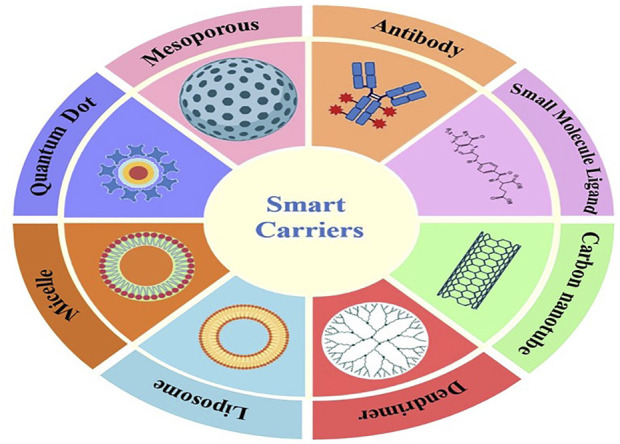
Some examples of Smart Drug Delivery Carriers.

Special carrier systems are required for cancer-targeted drug delivery applications. A SDDSs carrier is a special molecule, particle, composite, or system that can hold the drug, either through encapsulation or using a spacer. The TL is one of the most significant components for the successful delivery of drug payload in a SDDS. They segregate, transport, and hold drug payloads while delivering them to a specific targeted site. SDDSs require different carrier systems depending on the type of targeting mechanism. SDDSs carriers are specially designed vectors capable of encapsulating and/or bonding with a spacer moiety to keep the drug inside or on them (Figure 3). The medication delivery vehicle utilised must be non-toxic and non-immunogenic, stable, biocompatible, biodegradable, readily eliminated from the body, and unrecognizable by the host’s defence mechanisms. Other characteristics of drug carriers include high loading/encapsulation quantity with zero premature release of drug molecules, cell type or tissue specificity and site directing ability, and appropriate regulated release rate of drug molecules with an effective local concentration (Vallet-Regi et al., 2001). SDDSs based on carriers/TL provide benefits such as a larger surface-to-volume ratio, more reactive activity centres, more adsorption capacity, and other characteristics such as morphological preferences. The mechanism of control and drug secretion by these carriers at the target locations is very distinct and special. The reason is that the SDDS cleaves at first upon exposure to a particular stimulus, leading to continuous release for a long time afterwards (Rai et al., 2022).

#### 2.1.4 Therapeutic drug payload

SDDSs for targeted chemotherapy usually consist of the carrier, a cleavable linker, and the chemotherapeutic agent (Rana and Bhatnagar, 2021). The chemotherapeutic agent is chosen to be inactive in its conjugated form, which makes the SDDS a prodrug that is activated only in the tumor tissues.

**FIGURE 4:**
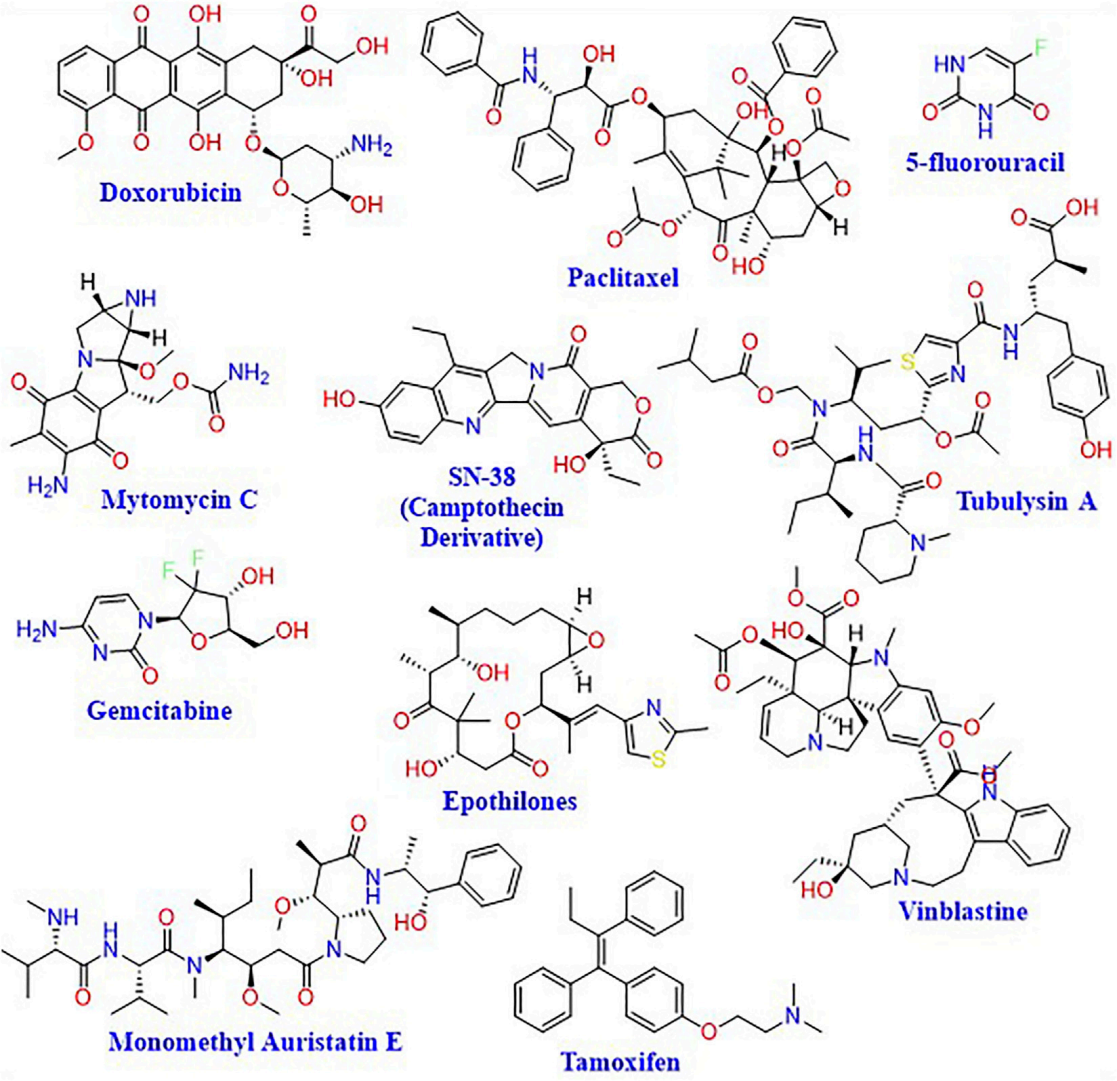
Chemical structures of representative cytotoxic agents used in SDDSs.

This allows the chemotherapeutic agent to exert its desired toxic activity on the cancer cells in a fast and effective manner (Figure 4). Drug delivery systems having the ability to attach targeted moieties can be given locally or systemically. The drug payload might be delivered either outside or within the target cells. Larger drug-delivery systems can provide high local drug concentrations, whereas smaller drug-delivery systems can be directly endocytosed. The precise architecture of the SDDSs allows drug payloads to be delivered to particular tissues in systemic administration. The main focus of SDDSs platform that the drug does not easily extravasate during blood circulation, but rather only releases at the sites where the drug carriers concentrate *via* an active or passive targeting approach (Liu et al., 2016).

## 3 mechanism of action of smart drug delivery system

**FIGURE 5:**
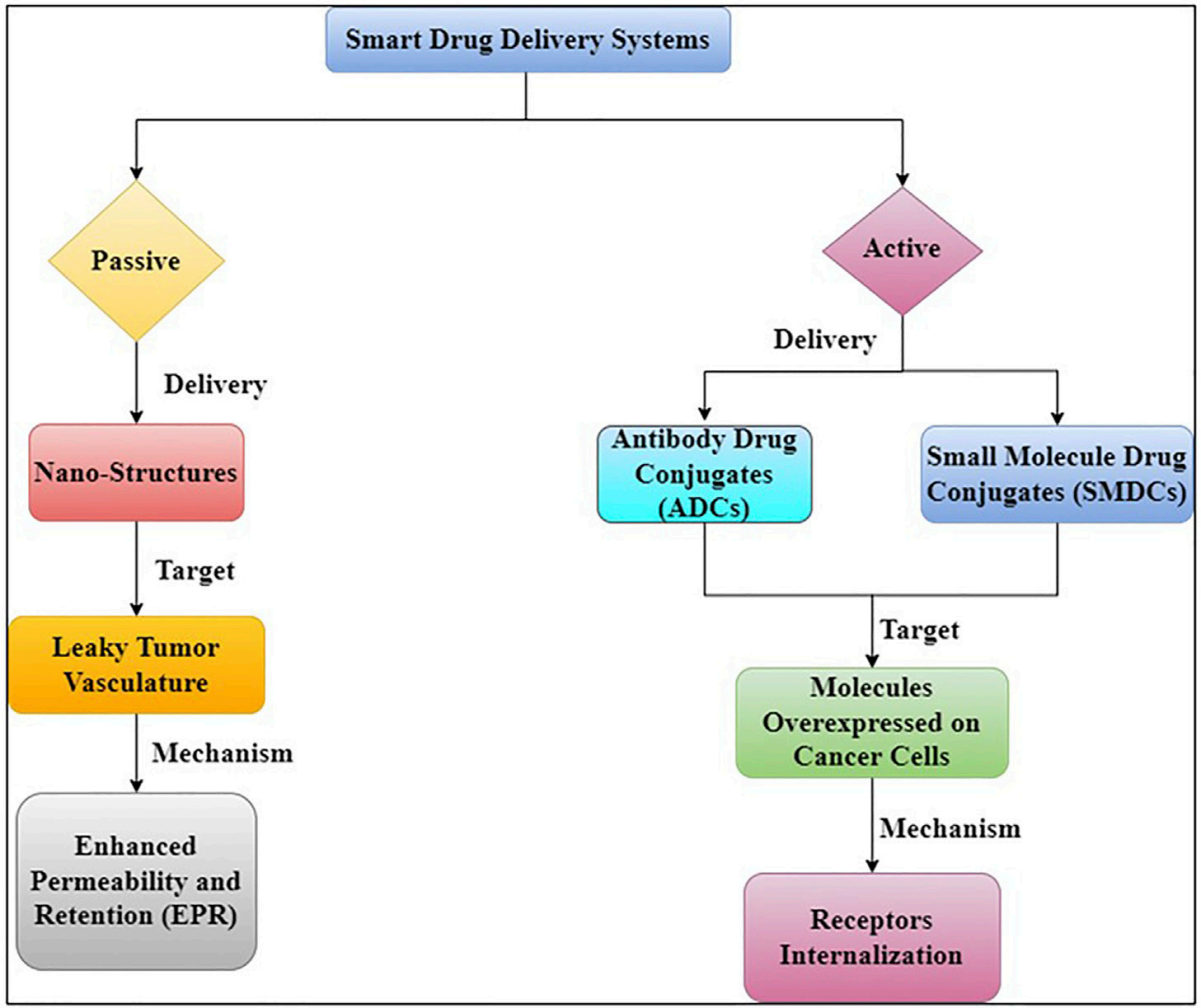
Different targeting strategies for anticancer therapeutics.

Early detection, location of the original tumour and metastases, killing cancer cells as effectively as possible while limiting harm to the patient (i.e., maximising therapeutic index), and high accumulation in tumour lesion are all important factors in cancer treatment (Kue et al., 2016). Drug targeting can be an effective strategy to address these challenges and overcome some of the drawbacks of non-targeted treatments. To deliver therapeutic payloads to tumour locations, there are two main mechanisms of drug targeting (Figure 5) (Torchilin, 2010).

Passive targeting relies on the use of large, generally polymeric molecules as carriers to increase permeability and retention. Targeting moieties such as ligands and antibodies are used in active targeting. These approaches differ from mechanistic or direct targeting options, which use monoclonal antibodies (mAbs) or small-molecule drugs to bind to surface proteins or interfere with elevated metabolic processes in cancer (Kue et al., 2016).

### 3.1 Active targeting delivery systems

**FIGURE 6:**
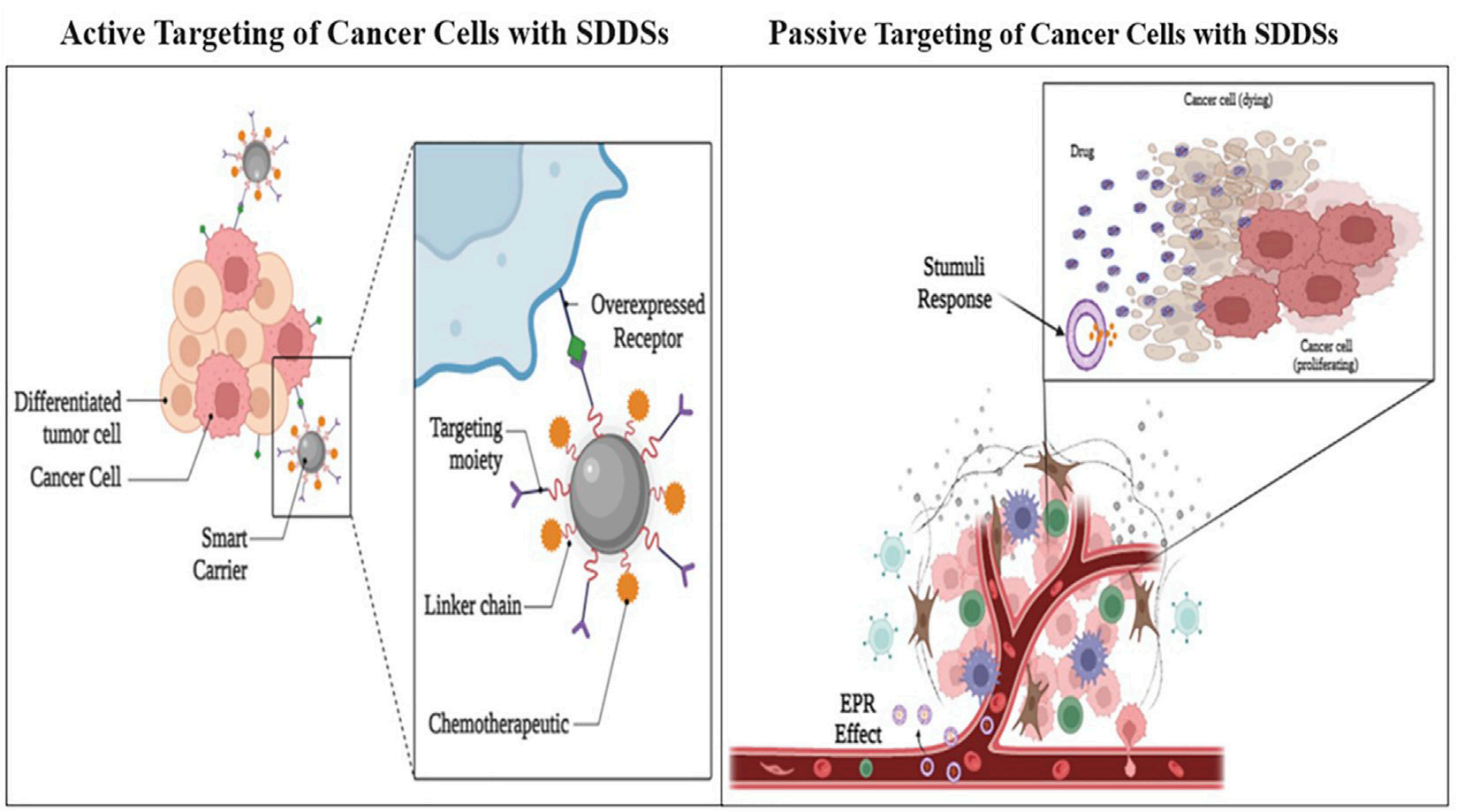
Active and Passive targeting delivery of drug payload by SDDSs.

Active targeting entails the identification of cancer cells, which leads to increased drug accumulation and cellular internalisation (Figure 6) (Kim et al., 2018). In active drug targeting, antibodies, antibody fragments, and peptides are linked to drugs and delivery systems to function as homing devices, allowing them to bind to receptor structures expressed at the target region. In terms of receptors on the cell surface and antigen expression, cancer cells vs healthy cells can be distinguished (e.g., Folate Receptors, transferrin’s and Prostate-Specific Membrane Antigen (PSMA)). Trans-membrane communication is facilitated by cell surface receptors, which are proteins anchored in the cell membrane. Active targeting refers to the employment of externally coupled targeting moieties to improve carrier distribution. Because a quickly developing tumour needs a wide range of minerals and vitamins, tumour cells overexpress several tumor-specific receptors. Nanoparticles are tethered with ligands such antibodies, peptides and folic acid that serve as targets that bind to those receptors, which may aid internalisation following engagement, to provide efficient tumor-specific drug delivery. G-protein-coupled receptors (GPCRs), integrins, folate receptors, transferrin receptors, epidermal growth factor receptor (EGFR), fibroblast growth factors (FGFRs), and sigma receptors are all used to target medicines to tumour tissues and microenvironments (Rana and Bhatnagar, 2021).

Antibodies have long been recognised to detect malignancies, particularly receptors or surface antigens with a high level of expression on cancer cells. In 1975, the first tumour antigen-targeting monoclonal antibody (mAb) was produced and since then, several mAbs have been FDA-approved for cancer therapy (Baah et al., 2021). Long-term administration of mAb-conjugated drug carriers is thought to create immunological memory against antibodies, although targeted treatment using mAb-conjugated drug carriers is regarded a key possibly curative approach (Kim et al., 2018). Antibody fragments or chimeric antibodies have the potential to significantly lower immunogenicity when compared to full antibodies (Singh et al., 2018). Cetuximab, a recombinant chimeric mAb with a murine variable region and a human constant region that has been successfully used to treat cancer by targeting the epidermal growth factor receptor (Singh et al., 2018). Dual targeting antibodies, which have two epitope binding sites and may respond with single or dual targets, are an emerging approach for improving tumour targeting capabilities (Kim et al., 2018). Trastuzumab, a humanised mAb for the treatment of HER2-positive breast cancer, was developed in 1998 (Piccart-Gebhart et al., 2005). Bevacizumab, a tumour angiogenesis inhibitor that binds to vascular endothelial growth factor, was authorised in 2004 for the treatment of colorectal cancer (VEGF) (Ferrara et al., 2004). Recent research has attempted to encapsulate chemotherapeutic medicines in nanoparticles and then functionalize the particle surface with mAbs to preserve targeted effectiveness. The nanoparticles’ absorption and cytotoxic efficacy are improved by conjugated antibodies (Sanchez-Moreno et al., 2018).

When metabolic activities are enhanced, transferrin receptors are overexpressed on cell surfaces (Shen et al., 2018). The primary route of cellular iron absorption *via* clathrin-coated pits, with subsequent traffic to endosomal compartments, has been shown to involve membrane transferrin receptor-mediated endocytosis of the complex of transferrin-bound iron and transferrin receptor (Tortorella and Karagiannis, 2014). Anti-tumor medicines, proteins, and therapeutic genes have all been effectively delivered through this absorption route (Sanchez-Moreno et al., 2018). Due to high iron needs, transferrin receptors have been found to be increased in malignant cancer cells, including those of bladder, brain, breast, and lung cancers, as well as lymphoma (Kim et al., 2018).

Aptamers are three-dimensional DNA or RNA sequences that are short and single-stranded. Aptamers are nucleic acid molecules that fold into complex 3D shapes that bind to specific targets, much like antibodies (Dunn et al., 2017). They’re gaining a lot of attention in clinical trials for a variety of reasons, including their prolonged storage life, narrow batch-to-batch differences, low immunogenicity, and the ability to make chemical modifications for improved stability, serum half-life extension, and targeted delivery (Ni et al., 2021). Aptamers are more stable ligands *in-vivo* than antibodies, as they are produced chemically *via in-vitro* selection, a simple and inexpensive process and the time required to generate aptamers is comparatively short. Unlike antibodies, aptamers do not require any specific biological systems for their production (Thiviyanathan and Gorenstein, 2012). According to Zhang et al., cell-based SELEX has a lot of potential since cancer cells may be targeted specifically without knowing the proteins expressed on their surfaces; hence, different aptamers can be produced to target different kinds of cancer (Zhang et al., 2020). Aptamers, have limitations, on the other hand as their affinity is lower than the one of antibodies. The most significant success of aptamers thus far has been the development of FDA-approved aptamers that can bind to VEGF, a protein involved in angiogenesis (Kaiser, 2006). The coupling of aptamers to drug-delivery nanoparticles resulted in better targeting, more effective treatments, and more selective diagnostics (Sanchez-Moreno et al., 2018).

Folate is a B9 vitamin that is water soluble and interacts with folate receptor to aid cellular uptake. The folate receptor has the advantage of having low expression in normal tissues, but it is strongly expressed by numerous malignancies, particularly cancers that afflict women, such as cervical, breast, and ovarian cancer (Rana and Bhatnagar, 2021). Folic acid binds 20 times more to tumour cells than it does to normal epithelial cells or fibroblasts. Folate conjugation has been a popular approach for targeting drug delivery systems due to these appealing features (Rana and Bhatnagar, 2021).

CTPs (cell targeting peptides) are peptides that are short and have been chemically synthesised from peptide libraries and utilised as targeting ligands (Vives et al., 2008). CTPs are less than 10 amino acids in length and are more stable than traditional antibodies (Dissanayake et al., 2017). The amino acid sequences of CTPs identify targets, and the best sequences for interacting with specific cancer cell surface receptors are important for target identification. The most well-studied CTP is the Arginylglycylaspartic acid (RGD) peptide, which has a high affinity for integrin receptors overexpressed on the surfaces of 21 different types of cancer cells (Sanchez-Moreno et al., 2018).

### 3.2 Passive targeting delivery systems

Passive targeting refers to the accumulation of a drug or drug-carrier system at a specific location, which can be caused by physicochemical and pharmacological variables (Eckmann et al., 2014). There are few universally applicable methods for targeting tumours and tumour cells due to the phenotypic diversity of malignant cells and tumours (or their organelles). The most important approach is since many cancerous cells and vascularized solid tumours, as well as some vascularized metastatic tumour nodules, have an enhanced permeability and retention (EPR) effect that can be used for antitumor drug “passive targeting” (Maeda et al., 2000). Because many solid tumours have a leaky vasculature and absent or limited lymphatic drainage, high molecular weight molecules (such as polymers) and small particles with a diameter of ∼20–500 nm accumulate within the tumour tissue (Ulbrich et al., 2016). This form of targeting is based on the pathophysiology of the disease and the characteristics of tumour tissues, which may encourage drug accumulation in target tissues, reducing non-specificity (Rabanel et al., 2012). The vasculature of tumours is thought to differ from that of surrounding tissue. In comparison to typical well-organized arteries, tumour angiogenesis has featured that aid drug retention, such as high vascular density and permeability, defective vascular architecture, and poor lymph drainage from tumour tissue interstitial spaces. The Enhanced permeability and retention effect (EPR) effect is used in passive targeting to detect cancer spots (Sanchez-Moreno et al., 2018). The accumulation rate of drug-loaded nanocarriers in a tumour is much higher than in normal tissue because to the leaky endothelium of the tumour vasculature (Hossen et al., 2019). The concentration of anti-cancer drugs in the tumour might be raised several times when compared to healthy bodily tissue using this EPR effect. The passive targeting of gelatin (typeB) –based NPs was extremely effective in the delivery of genes at tumour locations, according to Kommareddy et al. (Kommareddy and Samiji, 2007). Another study utilised gelatin (type B) for the creation of NP-based DDSs that included plasmid DNA (pDNA) (Kaul and Amiji, 2002). Encapsulating DNA with PEGylated gelatine NPs improved the efficiency of targeting pDNA-expressed green fluorescent proteins and -galactosidase *in vitro* as well as *in vivo*. PEGylated-gelatin NPs have also been utilised to focus on DNA moieties in lung carcinomas, suppressing tumour development and angiogenesis in breast cancer cells (Das et al., 2020; Mi, 2020).

## 4 Stimuli responsive smart drug delivery systems

**FIGURE 7:**
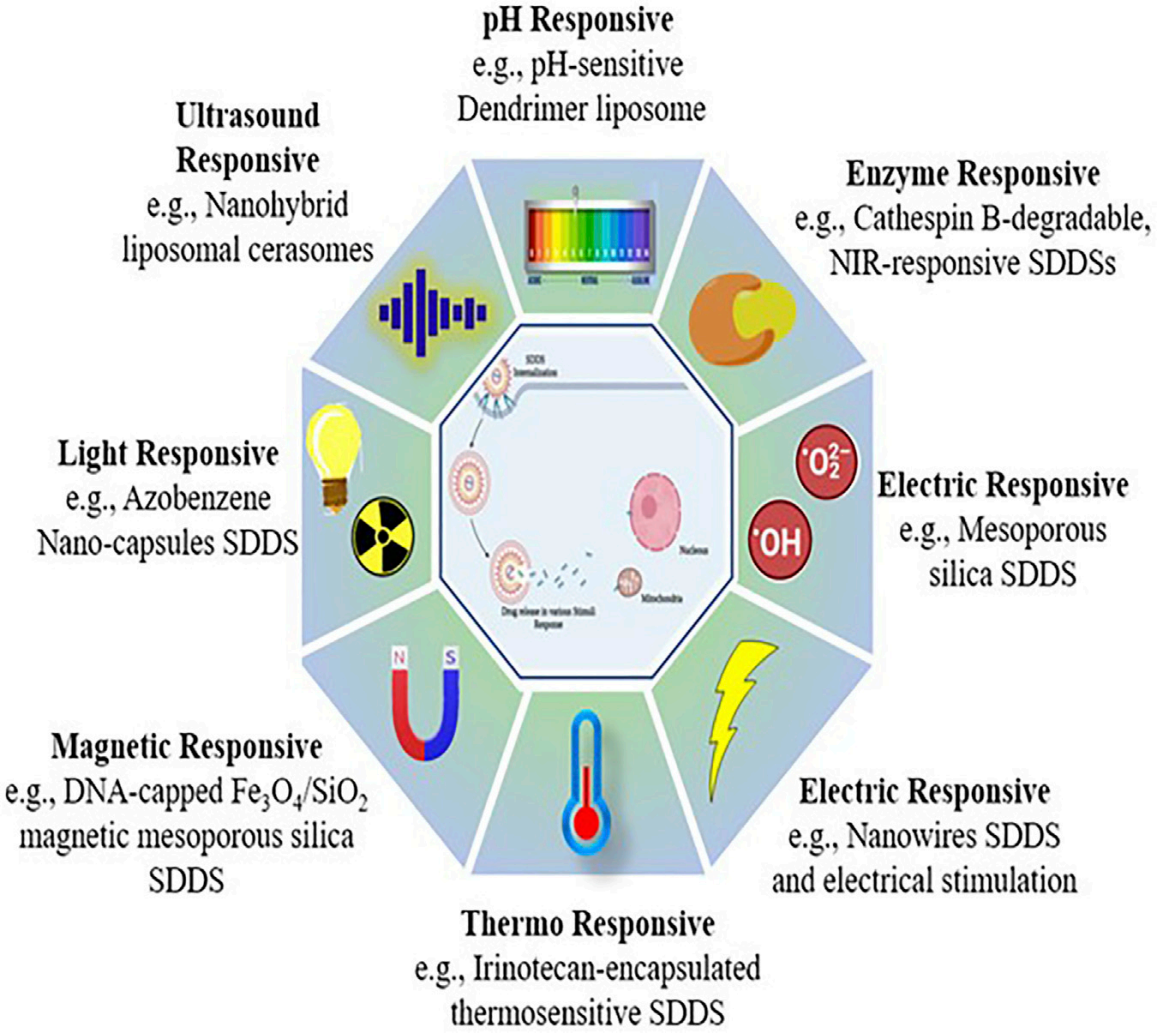
Stimuli responsive smart drug delivery.

The active drug can be released at the location in released under strict restraint systems in response to specific physical, chemical, or biological processes, some of which are triggered internally and some of them are induced externally (Sershen and West, 2002). Stimulus-based drug delivery techniques have showed a lot of promise in terms of successfully targeting active drug moieties. The first time thermo-sensitive liposomes were utilised for medication delivery was in 1978 (Mazzotta et al., 2018). Over the years, scientists have created and widely used stimuli-responsive biomaterials for regulated drug administration, culminating in the development of the area of stimuli-responsive polymers (Mi, 2020). As a result, they may be divided into two types of responsive DDS (Figure 7).

1. Exogeneous stimuli-responsive SDDSs (Open-loop system): Externally controlled systems, or pulsatile systems, are also known as open-loop systems. Magnets, temperature, ultrasound, electric effects, and in these systems, external triggers were employed to deliver the drug (Wen et al., 2018).
2. Endogenous stimuli-responsive SDDSs (Close-loop system): These are also known as self-regulating or responsive medication delivery systems. pH, enzyme-responsive drug delivery systems, and other internal triggers like redox-responsive drug delivery systems, *etc.*, controls the drug release from a closed loop control system (Wen et al., 2018). Drug release needs structural changes across the carrier or in specific layers or channels due to the fact that stimulation, according to SDDS (Mi, 2020). There are two types of stimuli: exogenous and endogenous. The utilisation of endogenous cues such as pH, glutathione (GSH), and certain enzymes allows for non-invasive, spatiotemporally regulated medication delivery (Mousavi et al., 2020). Different stimuli-based energy sources (light, ultrasonic, magnetic) that efficiently trigger drug release from nano cargos for effective delivery to specific locations (Table 1).

**TABLE 1:** Examples of Stimuli-Sensitive various smart-carriers developed for SDDS.

Stimulus	SDDSs	Smart Carrier/Ligand	Target	Drug Payload	Cancer	*In vitro* Cell lines	*In vivo* tumor Model	Ref
pH	Dox-loaded RGD-modified GQDs (Dox-RGD-GQDs)	RGD	α_v_β_3_ integrins receptors	Doxorubicin	Prostate cancer	DU-145, PC-3, and MC3T3-E1 cell lines	****	Qiu et al. (2015)
	HA/α-TOS@ZIF-8 nanoplatform	Hyaluronic acid	CD44 receptors	D-αTocopherol succinate (α-TOS)	Cervical cancer	HeLa cell line	Kunming mice	Sun et al. (2019)
	ATRAM-BSA-PLGA NPs	ATRAM	Membrane surface	Doxorubicin	Breast cancer, Cervical cancer, and human pancreatic carcinoma	MCF-7, HeLa, MIA PaCa-2 cells and mouse neuroblastoma Neuro-2a cell lines	Female C3H/HeJ mice	Palanikumar et al. (2020)
	TfR ligand (7pep; amino-acid sequence: HAIYPRH) conjugated micelle	TfR ligand (7pep; amino-acid sequence: HAIYPRH)	Transferrin receptors	Doxorubicin	Breast cancer	MCF-7/Adr cell line	Nude mice bearing drug-resistant MCF-7 xenografts (MCF-7/Adr)	Gao et al. (2017)
	DHA-GO-Tf	Transferrin	Transferrin receptors	Dihydroartemisinin	Breast cancer	Murine mammary tumor EMT6 Cell line	Balb/c female mice	Liu et al. (2015)
	Tri-Dox-FA-A-NPs	Folic acid and the AS1411 aptamer	Folate receptor and nucleolin receptor	Doxorubicin	Breast and pancreatic cancer	MCF-7, PANC-1 and L929 cell lines	****	Lale et al. (2014)
	D-Biotin/DOX-loaded mPEG-OAL/N-CQDs	D-Biotin	Biotin receptor	Doxorubicin	Cervical cancer	Hela cell line	****	Bao et al. (2019)
	FA-BSA-CAD	Folic acid	Folate receptor	Doxorubicin	Breast cancer, Hepatic cancer, and Lung cancer	MDA-MB-231, MCF-7, Bel-7402, HELF cancer cell lines	Kunming mice	Du et al. (2013)
	IgG1 (XE114)-vc-MMAE—ADC (+)	IgG1 (XE114) Monoclonal antibody	Carbonic Anhydrase IX (CAIX)antigen	Monomethyl Auristatin E	Human renal cell carcinoma	SKRC-52 cell lines	Female BALB/c nu/nu mice	Cazzamalli et al. (2018)
	AAZ- CA-IX-vc-MMAE SMDC	CAIX ligand	Carbonic Anhydrase IX (CAIX)	Monomethyl Auristatin E	Human renal cell carcinoma	SKRC-52 cell lines	Female BALB/c nu/nu mice	Cazzamalli et al. (2018)
	EC2220	Folic acid	Folate receptor	Vinca alkaloid	Squamous cell carcinoma, Lung cancer and Breast cancer	KB	Female BALB/c nu/nu mice	Leamon et al. (2006)
						M109, and 4T1 cell lines		
Redox	DOX-loaded HPAEG-AS1411 nanoparticles	Aptamer AS1411	Nucleolin receptor	Doxorubicin	Breast cancer	L929, MCF-7 cell lines	****	Zhuang et al. (2016)
	DOX-loaded star-PECLss-FA	Folic acid	Folate receptor	Doxorubicin	Breast cancer and Cervical cancer	HeLa, 4T1 cell lines	Female BALB/c mice	Shi et al. (2014)
	DOX@MSNs-S-S-Tf	Transferrin	Transferrin receptors	Doxorubicin	Hepatic cancer	Huh7 cell line	****	Chen et al. (2017)
	DOX@MSN-ss-GHA	Hyaluronic acid	CD44 receptor	Doxorubicin	Breast cancer and Cervical cancer	4T1 and HUVEC cell lines	female Balb/c mice	Chen et al. (2016b)
	Folate-Vinca Alkaloid Conjugate (EC145)	Folic acid	Folate receptor	Vinca alkaloid	Human nasopharyngeal carcinoma	KB, 4T1 cell lines	female nu/nu mice and female BALB/c mice	(Vlahov et al., 2006; Reddy et al., 2007)
	DOX@MSN-S-S-RGD	RGD	α_v_β_3_ integrins receptors	Doxorubicin	glioblastoma	U87 MG cell lines	****	Li et al. (2015)
	HA9.5-ss-PTX	Hyaluronic acid	CD44 receptor	Paclitaxel	Breast cancer and Skin cancer	MCF-7, B16F10 and VERO cell lines	Male BALB/c nude mice	Yin et al. (2015)
	Inotuzumab ozogamicin	Anti-CD22 mAb (G544, IgG4 isotype)	CD22 antigen	Calicheamicin	B-cell malignancy	Ramos (CRL-1923), Raji (CCL-86), Daudi (CCL-213), RL (CRL-2261), and HL-60 (CCL-240) cell lines	Female, athymic BALB/c nu/nu (nude) mice	DiJoseph et al. (2004)
	DOX@MSNs-CAIX	Anti-carbonic anhydrase IX antibody (A-CAIX Ab)	Carbonic Anhydrase IX (CAIX)antigen	Doxorubicin	Breast cancer	4T1-Luc (Luciferase), Mef cells (mouse embryo fibroblast) cell lines	BALB/C mice	Chen et al. (2020a)
Enzyme	PTX-loaded PEG-GPLGVRGDG-PDLLA nanoparticle	RGD	α_v_β_3_ integrins receptors	Paclitaxel	Breast cancer	4T1 cell line	Female CD-1 (ICR) mice	Ke et al. (2017)
	MSNs-Peptide-BSA-LA@DOX	Lactobionic acid	asialoglycoprotein receptor (ASGP-R)	Doxorubicin	Hepatocellular carcinoma	BEL7402 cell lines	Balb/c mice	Bansal et al. (2016)
	Ac-La-G (4)-PAMAM-FITC dendrimer loaded with sorafenib	Lactobionic acid	asialoglycoprotein receptor (ASGP-R)	Sorafenib	Hepatocellular carcinoma	HepG-2 and HLE cell lines	****	Iacobazzi et al. (2017)
	FA-GFLG-SN38	Folic acid	Folate receptor	SN38	Cervical cancer, Lung cancer and liver cancer	HeLa, Siha	****	Jin et al. (2020)
						A549, and SK-Hep-1 cell lines		
	FA-GFLG-MMC	Folic acid	Folate receptor	Mitomycin C (MMC)	Cervical cancer and Lung cancer	HeLa, SiHa, PC9, A549, and 16HBE cell lines	****	Xu et al. (2020)
	FA-conjugated CDDP-loaded Mal-PEG-b-PLG-FITC vesicles	Folic acid	Folate receptor	cisplatin (CDDP)	Cervical cancer	HeLa and NIH-3T3 cell lines	****	Shirbin et al. (2015)
	Hyaluronic acid coating caspase 3 loaded drug nanoparticles	Hyaluronic acid	CD44 receptor	Paclitaxel	Breast cancer	4T1 and MCF-7 cell lines	MCF-7 tumor-bearing Balb/C nude mice	Xin et al. (2018)
	PEGylated lysine peptide dendrimer-gemcitabine conjugate	****	****	Gemcitabine	Breast cancer	4T1 and COS-7 cell lines	Female BALB/C mice	Zhang et al. (2017)
	Gemcitabine (GEM) nanovectors (RGD-GEM-GELG- CdSe/ZnS)	CycloRGD	α_v_β_3_ integrins receptors	Gemcitabine	Pancreatic cancer	BxPC-3 cell lines	BxPC-3 xenograft models in nude mice	Han et al. (2017)
	Folate bound poly (ethylene glycol)-distearoylphosphatidylethanolamine (FA-PEG-DSPE)	Folic acid	Folate receptor	Paclitaxel	Breast cancer	MDA-MB-231, MDA-MB-468, BT-20, and T47-D cell lines	Female athymic nude mice	Satsangi et al. (2015)
Light	Photocaged folate nanoconjugates	Folic acid	Folate receptor	Paclitaxel	Cervical cancer	KB cell lines	****	Fan et al. (2012)
	FA adsorbed PC_12_NB polymersomes (PC_12_NB + DOX + FA + hn)	Folic acid	Folate receptor	Doxorubicin	Cervical cancer	HeLa cell lines	****	Zhou et al. (2020)
	Folate-targeted gold nanorods (AuNRs@PHEA-EDA-FA)	Folic acid	Folate receptor	Nutlin	Human osteosarcoma and Lung Cancer	U2OS, 16HBE and HDFa cell lines	****	Li Volsi et al. (2017)
	HMS/C18/PRMS-FA	Folic acid	Folate receptor	Doxorubicin	Cervical cancer and Lung Cancer	KB and A549 cell lines	****	Xing et al. (2014)
	AuNPs with the folate PEG-SH and PSS (Au@folate-PEG-PSS)	Folic acid	Folate receptor	Doxorubicin	Breast cancer	MCF-7 and MDA-MB-231 cell lines	****	Banu et al. (2015)
	GNR-embedded Diblock copolymer [PEG-bpoly (2-hydroxyethyl	Folic acid	Folate receptor	GW627368X	Cervical cancer	SiHa, ME180, HaCat, and 3T3 cell lines	S180 bearing Swiss albino mice	Parida et al. (2017)
	acrylate)–lipoic acid–folic							
	acid] micelles							
	Dopamine-adipic acid dihydrazide-hyaluronic	Folic acid/Hyaluronic acid	Folate/CD44 receptor	Doxorubicin	Breast cancer	MCF-7 cell lines	female BALB/c nude mice	Xu et al. (2017b)
	acid trifuncitionalized gold							
	nanorod (GNRs-HA-FA-DOX)							
	DOX-EGCG/DPA-FA NPs	Folic acid	Folate receptor	Doxorubicin	Breast cancer	4T1 cell line	4T1 tumor-bearing BALB/c mouse model	Fan et al. (2021)
	DOX-MUCNP@C18@PSMN-FA	Folic acid	Folate receptor	Doxorubicin	Cervical cancer and Lung cancer	KB, A549 and Beas2B cell lines	KB tumor bearing nude mice	Xing et al. (2015)
	PDA-RGDC/DOX	Arginine glycine-aspartic-cysteine acid (RGDC) peptide	α_v_β_3_ integrins receptors	Doxorubicin	Cervical cancer	HeLa cell line	HeLa tumor-bearing BALB/c mouse model	Li et al. (2017a)
	Biotin-PEG-GNR-DNA/DOX (BPGDD)	Biotin	Biotin receptors	Doxorubicin	Breast cancer	MCF-7 and MCF-7/ADR cell lines	****	Zhang et al. (2016)
	PB@PDA@PEG-FA-DOX	Folic acid	Folate receptor	Doxorubicin	Cervical cancer	HeLa and HL-7702 cell lines	Hela tumor-bearing nude mice	Lin et al. (2019)
Ultrasound	Paclitaxel-liposome–microbubble complexes (PLMC)	Biotin	Biotin receptors	Paclitaxel	Breast cancer	4T1 cell lines	4T1 tumor-bearing female BALB/c mice model	Yan et al. (2013)
	Paclitaxel loaded hyaluronic acid targeted liposome (HA-Lipo/PTX)	Hyaluronic acid	CD44 receptor	Paclitaxel	Breast cancer	4T1 and T47D cell lines	4T1 tumor-bearing female BALB/c mice model	Ravar et al. (2016)
	Microbubble-liposome complex (IRMB-OxLipo)	Biotin	Biotin receptors	FOLFIRINOX (Irinotecan and Oxaliplatin)	Pancreatic cancer	Panc-01 3D spheroid	BxPC-3 human xenograft murine models	Gao et al. (2020)
	PTX@FA--CD/H-MSN (DESN)	Folic acid	Folate receptor	Paclitaxel	Breast cancer	4T1 cell lines	4T1 tumor-bearing female BALB/c nude mice model	Wang et al. (2018)
	A10-3.2/siCAT-1/3WJ-NDs	A10-3.2 aptamer	Prostate specific membrane antigen (PSMA)	siCAT-1 (siRNA)	Prostate cancer	22RV1, PC-3 and 16HBE	****	Guo et al. (2022)
	Span–PEG with FA–CNT–PTX	Folic acid	Folate receptor	Paclitaxel	Breast cancer	MCF-7 cell lines	MCF-7 tumor-bearing mice model	Zhang et al. (2019)
	TRAIL-Dox-Nanoshards	Tumor necrosis factor-related apoptosis inducing ligand (TRAIL)	TRAIL–receptor	Doxorubicin	Breast cancer	MDA-MB-231, TRAIL-resistant MCF7 and MCF-12A	****	Jablonowski et al. (2018)
	ALN/FA-decorated PTX-loaded nanoparticles	Folic acid	Folate receptor	Paclitaxel	Breast cancer	4T1 cell lines	4T1 tumor-bearing female BALB/c nude mice model	Chen et al. (2020b)
	ANP-D/P	Angiopep-2	Lipoprotein receptor-related protein (LRP)	Doxorubicin	Glioblastoma	U87 MG and BCEC cell lines	U87 MG tumor-bearing female BALB/c nude mice model	Luo et al. (2017)
	LHRH-ELP-DOX	LHRH	Luteinizing hormone releasing hormone (LHRH) receptor	Doxorubicin	Breast cancer	MCF-7 and MCF-7/ADR cell lines	MCF-7/ADR tumor-bearing female BALB/c nude mice model	Wang et al. (2017)
Magnetic	DOX-FA-MN-MWCNTs	Folic acid	Folate receptor	Doxorubicin	Glioblastoma	U87 cell lines	****	Lu et al. (2012)
	Fe3O4@OCMC@IRMOF-3/FA	Folic acid	Folate receptor	Doxorubicin	Cervical cancer	HeLa cell line	****	Chowdhuri et al. (2016)
	DOX−SPION− (P(NIPAAm-coAAm)-b-PCL) micelles	Integrin β4 antibody	A9 antigen	Doxorubicin	Head and Neck cancer	SQ20B cell line	****	Kim et al. (2013)
	MSCN-PEG-HB5/DOX	HB5 aptamer	HER2 receptor	Doxorubicin	Breast cancer	SK-BR-3 cell lines	SK-BR-3 tumor-bearing female BALB/c nude mice model	Wang et al. (2015)
	MagO2MB-RB-Gem conjugate	Biotin	Biotin receptors	Gemcitabine	Pancreatic cancer	BxPC-3 and Mia-PaCa-2 cell lines	Xenograft ectopic BxPC-3 tumours in SCID mice	Beguin et al. (2020)
	HER2-paclitaxel-GMO-MNPs	HER2 antibody	HER2 receptor	Paclitaxel	Breast cancer	MCF-7	****	Dilnawaz et al. (2010)
	Dox loaded-CD105-conjugated SWCNTs	Mouse Endoglin/CD105 mab	Endoglin/CD105	Doxorubicin	Breast cancer	4T1-Luc2 cell line	4T1-Luc2 tumor-bearing female BALB/c mice	Al Faraj et al. (2015)
	Casein-CFNP-CNA-BT	Biotin	Biotin receptors	Cinnamaldehyde	Lung cancer	L929 and A549 cell lines	****	Purushothaman et al. (2021)
	DGNP Loaded and Folate	Folic acid	Folate receptor	Doxorubicin	ovarian	A2780, OVCAR3 and SKOV3 cell lines	CD-1 female nude mice	Ak et al. (2018)
	Attached Erythrocyte							
	Vesicles (FVzDGNP)							
	PFH/DOX@PLGA/Fe3O4-FA	Folic acid	Folate receptor	Doxorubicin	liver cancer	Bel-7402 cells, SKOV-3 cells, and MB-231 cell lines	Bel-7402 tumor-bearing female nude mice	Tang et al. (2018)

### 4.1 pH responsive stimulus

pH is one of the most commonly used triggers for drug release because of the significant pH difference seen at the cellular level between the cytosol (7.4), the Golgi apparatus (6.40), the endosome (5.5–6.0), and the lysosome (4.5–5.5) of cancer cells and in the tumour microenvironment (Mi, 2020). In general, the pH of cytoplasm, blood, and normal tissues is around 7.0 to 7.4, while endosomal/lysosomal organelles have a pH of 6 to 4, and the tumour microenvironment has a pH of 6.5–6.8 (Mi, 2020). The use of polymers with weak acids (e.g., carboxylic acid) or bases (e.g., primary and tertiary amines) groups is used to create pH-responsive systems that produce rapid changes in ionisation at the appropriate pH. The pH responsiveness of the polymer may be readily tweaked by changing the type of the co-monomers employed to make it (Darvin et al., 2019). A pH-responsive medication delivery system may be created by hydrazone bonding an anticancer agent to carriers or targeting ligands. A medication delivery system like this reacts to acidity inside tumour cells and releases drugs in a regulated manner. Following this technique, Du et al. developed PCC-Hyd-DOXDA, a custom-made dual pH-triggered polymer drug attached system. PCC-Hyd-DOX-DA has been found to be easily absorbed by MDA-MB-231 tumour cells at pH 6.8, whereas absorption at pH 7.4 is negligible (Du et al., 2011). The polyacidic pH-responsive system includes polyacrylic acid (PAAc) and polymethacrylic acid (PMAAc) (Chen et al., 2016a). The invention of a pH-triggered auto-fluorescent polymeric nanoplatform for the delivery of non-fluorescent aromatic nitrogen mustard chlorambucil (CBL) to cancer tumours was reported by Saha et al. (Saha et al., 2019). In another study, Zhang and others incorporated doxorubicin and dextran with a hydrazone linker, targeting hepatocytes with folate (Zhang et al., 2015). While pH is widely utilised in smart medicine administration, it should be combined with other stimuli such as temperature or redox to achieve extremely exact and precise release at the target locations. The use of acidic pH as a tumour microenvironment trigger has certain drawbacks. To begin with, the acid pH in perivascular areas is often remote from the blood flow, resulting in a lack of reaction of nanoparticles. In addition, pH changes in healthy tissues and malignant tissues are frequently similar (Pan et al., 2012; Cheng R. et al., 2013). For regulated release of doxorubicin, Nikravan et al. created a pH sensitive cross-linked nanoparticle system generated from various molar ratios of poly (acrylic acid) (PAA) and ethylene glycol dimethacrylate. With increasing cross-linking degrees, the pH-responsive behaviour of this nanocarrier was less effective. At pH levels of 1.2, 5.3, and 7.4, the release of the model drug doxorubicin was investigated (Dhanasekaran, 2015).

### 4.2 Redox responsive stimulus

The redox-sensitive drug delivery system has sparked a lot of attention in the field of therapeutic strategies, because of its close ties to a variety of diseases, and it is being investigated a lot (Mura et al., 2013). Additionally, the redox-sensitive delivery system has the benefit of drug release within the cancer cell. Redox hemostasis is a crucial process for cell survival that involves glycolysis, glutathione synthesis, fatty acid oxidation, and glutaminolysis (Panieri and Santoro, 2016). However, in tumour cells, dysregulated redox hemostasis resulted in a shift in redox balance and an increase in ROS levels. An increase in ROS levels was caused by mitochondrial dysfunction, overexpression of NADPH oxidases, and changes in antioxidant enzymes (Arcucci et al., 2016). The redox potential in microenvironments tends to vary depending on the tissue, which may be exploited to develop redox-responsive delivery systems. The reducing environment of tumour cells is largely determined by NADPH/NADP+ and glutathione (GSH, GSH/GSSG), both of which have different reduction potentials and capacities (Wu et al., 2004). GSH levels differ between normal and cancerous cells. It ranges from 2 to 20 μM in blood and normal extracellular matrices, whereas it ranges from 2 to 10 mM in cancer cells which is 100- to 500- fold higher than the normal ranges (Liu et al., 2016). To produce redox-responsive carriers, the disulfide bond has been proven to be the major redox-sensitive linker (Liu et al., 2017). GSH levels in intracellular compartments (cytosol, mitochondria, and nucleus) are two to three orders of magnitude greater (2–10 mM) than in external fluids (2–20 mM). As a result, GSH is a well-known intracellular molecule that may be used to induce drug release within cells (Indermun et al., 2018). Many studies on the redox responsiveness of disulfide bonds are currently in progress, and diselenide bonds are also getting a lot of attention as well. Diselenide redox-sensitive bonds delivery systems are similarly sensitive to reduction and have characteristics similar to disulfide connections. Diselenide bonds can be used to create a more sensitive redox-responsive delivery system in tumour therapy because their bond energies are lower than S–S bonds (Se–Se 172 kJ/mol; C–Se 244 kJ/mol; S–S 268 kJ/mol) (Guo et al., 2018). Gang Cheng et al. synthesised the polycationic carrier OEI_800_-SeSe_x_ by adding the active ester containing diselenide bonds to the branched oligoethyleneimine 800 Da (OEI_800_) (Cheng et al., 2012). The ability of SDDSs to respond to reactive oxygen or nitrogen species (ROS or RNS) is still barely explored (Alvarez-Lorenzo and Concheiro, 2014). The main contributors to the intra- and extracellular redox potential associated with stress conditions, signalling cascades, diabetes, hypertension, atherosclerosis, or cancer are ROS such as hydrogen peroxide, superoxide, or OH radicals (Lallana and Tirelli, 2013). Oxidation-responsive SDDSs are a subset of redox-sensitive drug delivery systems that rely on reactive oxygen species (Torres et al.), primarily H_2_O_2_ and OH radicals (Darvin et al., 2019). A redox-sensitive polymeric nanoparticle for tumor-targeted medication delivery was described by Cho et al. The paclitaxel-incorporated nanoparticle was prepared using a redox-responsive biodegradable polymer that was capable of delivering paclitaxel in response to a reduction process (Das et al., 2020).

### 4.3 Enzyme responsive stimulus

Due of its distinct benefits, such as substrate, specificity and excellent selectivity under moderate circumstances, enzymes employed as triggers in the construction of SDDSs have been a growing topic in recent years (Liu et al., 2016). Many enzymes have been put to work to enhance medication transport to cancer cells, including lipase, protease, trypsin, glycosidase, phospholipase, oxidoreductase, and others (De La Rica et al., 2012). The drugs will be released at the target locations by site-specific enzymatic cleavage by smart carriers/ligands bearing drug payload linked/conjugated to them *via* encapsulation or covalent bonding. The drug-release mechanism is triggered by several enzymes (Darvin et al., 2019). Proteases which degrade protein and peptides, a fantastic alternative for releasing medicines from liposomes (Hossen et al., 2019). Radhakrishnan et al. developed hollow nanocarriers triggered by the trypsin/hyaluronidase enzyme to deliver anticancer agents intracellularly (Radhakrishnan et al., 2014).

The phospholipase A2 (PLA2) enzyme is used to release medicines or expose target ligands from SDDSs that use liposomes or small unilamellar vesicles (SUVs) (Sanchez-Moreno et al., 2018). With the presence of Cathepsin B, an intracellular cysteine protease that was particularly overexpressed in tumour locations, the H-Phe-Lys-OH peptide could be broken. Hollow nanocarriers activated by the trypsin/hyaluronidase enzyme to deliver anticancer drugs intracellularly. MMPs (matrix metalloproteases) are a zinc-dependent family of endopeptidases that are well-known for their role in cancer prognosis (Liu et al., 2017) and have been extensively studied for drug delivery and imaging applications (Kessenbrock et al., 2010). Zhu et al. Developed MMP2-sensitive; PEG lipid conjugated liposomes with anti-nucleosome monoclonal antibodies modified on their surface to improve cancer targeting (Zhu et al., 2012). In another study, Chen et al. manufactured multifunctional poly (ethylene glycol)- blocked-poly (l-lysine) Biotin 6-maleimido-caproic acid (Biotin-PEG-b-PLL (Mal)-peptide) polymeric micelles enclosing doxorubicin to improve cancer cell uptake by endocytosis (Chen WH. et al., 2015). Despite its utility, enzyme responsive SDDSs lacks precise control over the system’s initial response time.

### 4.4 Light responsive stimulus

Light-responsive SDDSs have received much interest as a way to take advantage of either daily and seasonal exposure to natural solar irradiation or artificial sources of electromagnetic radiation with very specific wavelengths between 2500nm and 380 nm (Alvarez-Lorenzo and Concheiro, 2014). These photo-responsive drug delivery systems offer many advantages over other stimuli-responsive formulations for drug delivery because photochemical processes do not require additional reagents or catalysts, and the majority of by-products, if any, are harmless (Pan et al., 2021). Photosensitive carriers light-responsive smart drug delivery devices have an on/off drug release mechanism in response to irradiation stimulation. A photosensitive biomaterial is generally conjugated or encapsulated to a therapeutic agent in such SDDSs. The photosensitive material absorbs light (photons), which causes a conformational change in these smart-carriers, dramatically altering their structure and allowing the encapsulated/conjugated agent to be released at the desired site in a spatio-temporal controlled manner (Sanchis et al., 2019; Pan et al., 2021). UV and visible light can cause medication release from formulations that are applied to the skin or circulate through blood vessels near the body’s surface (e.g., eye structures). Drug release is usually initiated by reversible or irreversible photo-induced structural changes in smart-carriers. Photo-cleavable bonds can be used to conjugate medicines for light sensitive release (Alvarez-Lorenzo and Concheiro, 2014). For example, doxorubicin-encapsulated poly (lactic-co-glycolic acid) (PLGA)matrix particles with a gold over-layer, NIR-triggered release was observed. When cancer cells were exposed to NIR light, doxorubicin was released abruptly, resulting in high cancer cell toxicity and tissue ablation (Zhu et al., 2012). Specifically, carbon nanotubes and graphene nanoparticles (GNPs) are excellent candidates for light-triggered stimuli, in particular, the near-infrared (Prasanna et al.) range (Hossen et al., 2019). In order to destroy cancer cells, metallic nanocarriers are capable of absorbing light and convert it to heat (Alvarez-Lorenzo and Concheiro, 2014).

Azobenzene and its derivative-based nanocarriers are frequently utilised to regulate drug delivery at its target by using ultraviolet–visible light and/or visible light to facilitate structural change and drug release (Yan et al., 2012). By encapsulating doxorubicin and ammonium bicarbonate inside nanocarriers, Chen et al. created a bubble-generating thermo-responsive liposomal system. Ammonium bicarbonate decomposes at high temperatures, releasing carbon dioxide bubbles that generate permeable holes in the lipid bilayer of liposomal nanocarriers, allowing the loaded medication doxorubicin to be released quickly (Chen et al., 2013).

### 4.5 Ultrasound responsive stimulus

Ultrasound is a type of high-frequency sound wave that may have an impact on carriers used for controlled drug release at diseased sites (i.e., tumors). The ultrasound intensity could be adjusted for various applications. At low ultrasound frequencies (less than 20 kHz), it could be used for imaging, as well as disrupting smart-carriers to release cargos or increasing the permeability of cancer cell membranes at high ultrasound frequencies (greater than 20 kHz) (Mi, 2020). Ultrasound has become quite popular as a stimulus in clinical investigations because to its various benefits, including intrinsic tissue penetration, improved spatiotemporal control, and increased safety. Ultrasound has recently been popular in clinics as a diagnostic and therapeutic technique (Mi, 2020). The invention of nanocarriers with ultrasonic sensitivities for ultrasonography has expanded ultrasound procedures to become a unique and successful tool for capturing drug carriers and triggering drug release at the target locations by adjusting the ultrasound frequency, duty cycles, and exposure duration (Liu et al., 2016). Kruskal et al. used a nanocarrier-DOX-encapsulated delivery technique, followed by ultrasonic tumour irradiation, to accomplish tumour targeting, resulting in the drug’s systemic distribution. Wang et al. created amphiphile segments with ultrasound-sensitive oxyl-alkylhydroxylamine(-oa) linkages. To improve medication transport to hepatocellular carcinoma cells, hydrophobic DOX was encased between the hydrophobic amphiphile portion (Mi, 2020). Jung et al. created dual-functional Gd(III)-DOTA-modified sono-sensitive liposomes for doxorubicin administration and magnetic resonance imaging acquisition (Jung et al., 2012). In the realm of cancer treatment, ultrasonic therapy has been utilised in combination with micelles. Husseini et al. (Bulbake et al., 2017) examined the release of doxorubicin from Pluronic P105 micelles at various ultrasonic frequencies.

### 4.6 Magnetic responsive stimulus

Magnetic-responsive drug delivery systems offer a non-invasive method of controlling the carriers’ spatiotemporal proximity to their targets. The use of magnetic particles for the delivery of anti-cancer drugs or antibodies to organs or tissues altered by disease has become an active and appealing field of research since the pioneering idea proposed by Freeman et al. (Freeman et al., 1960) that fine iron particles could be transported through the vascular system and concentrated at a particular point in the body with the aid of a magnetic field (Estelrich et al., 2015). This aids the device in releasing payloads under programmed external magnetic field exposure. MNPs (magnetic nanoparticles) have an abundance of active sites for bio molecule conjugation, allowing for accurate design and engineering to achieve their intended smart functions by applying a localised external magnetic field, such as long-term circulation in the bloodstream, target specificity to lesion tissues, and therapeutic delivery (Darvin et al., 2019). The most widely used core/shell magnetic nanoparticle has a wide range of magnetic properties. The drug is combined with a pharmaceutically stable ferromagnetic carrier in this complex. There are a number of ways to create magnetic-responsive systems, such using nanoparticles, or magneto-liposomes (Madaan et al., 2014). Jiang et al. created magnetically tunable BSA (Fe_3_O_4_/BSA) particles coated with negatively charged iron oxide nanoparticles. The release of these particles from bone marrow mesenchymal stem cells, where they were internalised with the help of an external magnetic field, was delayed (Freeman et al., 1960). Li et al. created a magneto-thermally responsive nanocarrier/doxorubicin (MTRN/Dox) using Mn-Zn containing ferrite magnetic nanoparticles (MZF-MNPs) to form a thermosensitive copolymer coating with absorbed chemotherapeutic combined with the magnetothermal effect of MZF-MNPs to allow controlled drug release at the tumour site under an alternating magnetic field (AMF) (Li et al., 2018). When compared to free Dox and MTRN/Dox treatment without the use of an AMF, the authors found that magnetic targeting of MTRN/Dox increased accumulation in tumour tissues and that AMF treatment was required for MTRN/Dox increased cytotoxicity. The MTRN/Dox with combined magnetic targeting and AMF treatment showed the greatest tumour volume reduction compared to the MTRN/Dox with only magnetic targeting or AMF treatments after injection into nude mice bearing tumours, indicating that it has potential as a liver cancer therapy. Fang, Xiuqi et al. developed a highly controllable process of Carbon Encapsulated Magnetic Nanoparticles (CEMNs) synthesis in arc discharge plasma. With an external magnetic field, CEMNs have been made more controllable with respect to both their size distribution and purity and with an external magnetic field, CEMNs have been made more controllable with respect to both their size distribution and purity. For the purpose of assessing the potential for CEMNs to be used in biomedicine, the human breast cancer cell line MDA-MB-231 was used to determine the cytotoxicity of CEMNs. Based on this finding, it is concluded that specific CEMN dosages can be utilized in biomedical settings such as MRI, cell migration control, hyperthermia, and medication administration (Fang et al., 2018).

## 5 Smart drug delivery systems using smart nano-carriers in cancer therapy

**FIGURE 8:**
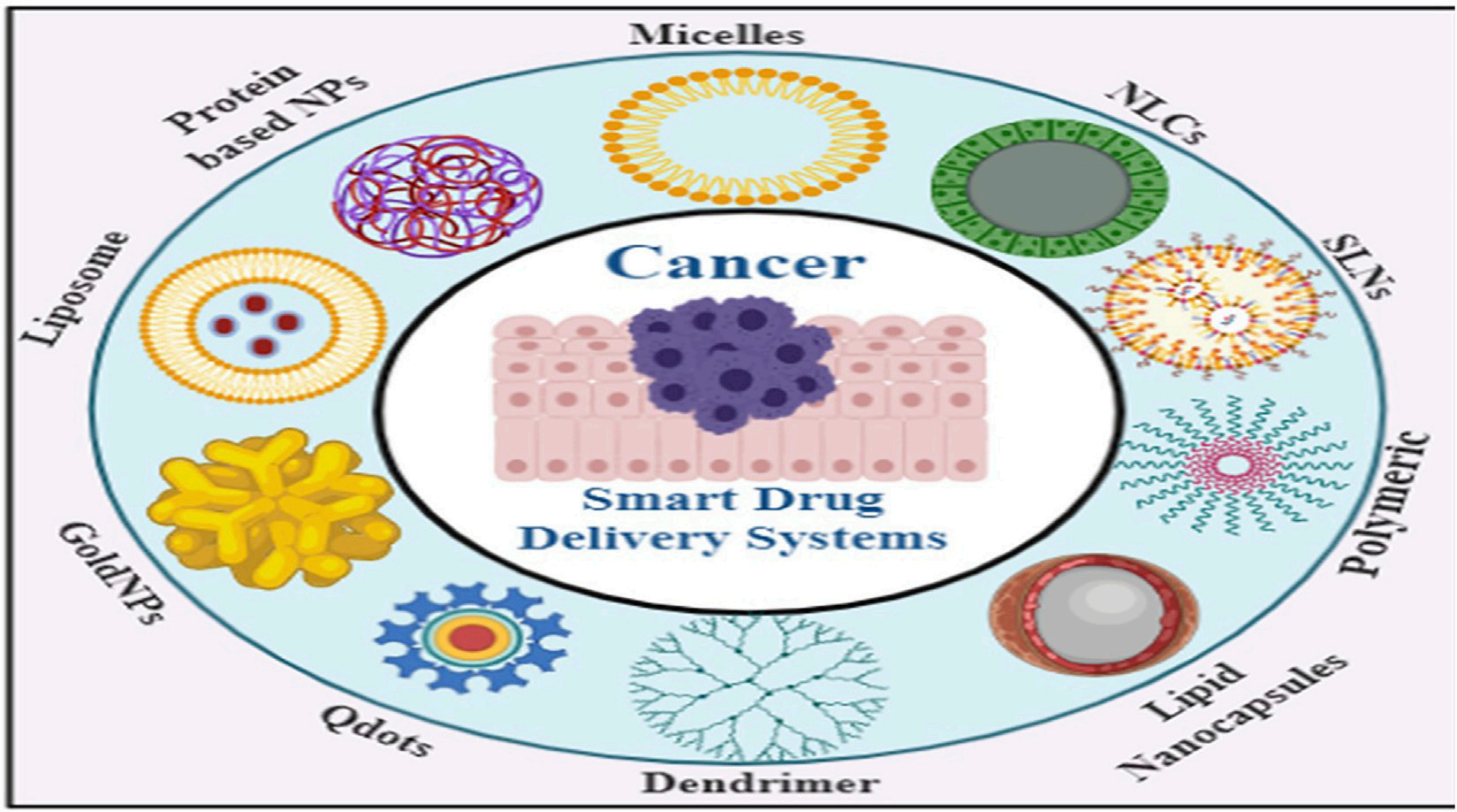
Smart nanocarriers used in SDDSs.

Nanotechnology is a cutting-edge, innovative, and promising method of delivering a drug payload to tumour tissue. Nanoparticles (NPs), which range in size from 1 to 100 nm, can reveal both physical and chemical properties; are more likely to be accumulated in solid tumors by passively extravasation from the hyperpermeable tumor blood vasculature (Cabral et al., 2011). Nanoparticle delivery systems are broadly evaluated preclinically with other nanoparticle-constructed formulations and technologies that have been used so far in the clinic setup (Peer et al., 2007; Shi et al., 2010; Wicki et al., 2015; Navya et al., 2018; Murugan et al., 2021). There are two types of therapeutic and diagnostic nanoparticles: [a] inorganic nanoparticles (such as gold, silica, and iron oxide) and [b] organic nanoparticles (e.g., polymeric, liposomes, and micelles) (Figure 8) (Murugan et al., 2021). Conventional nanocarriers are unable to transport and release drugs in the desired concentration at the targeted site when stimulated externally or internally. They must be improvised or functionalized to make them smart (Lee et al., 2015). The following qualities should be present in smart nanocarriers. To begin, smart nanocarriers should avoid the immune system’s cleaning process. Second, they should only be gathered at the targeted site. Third, upon external or internal stimulation, the intelligent nanocarrier should release the cargo at the correct focusing on the targeting site (Hossen et al., 2019). Finally, they must supply chemotherapeutics as well as other things for example, genetic materials and imaging agents, and other similar compounds (Peer et al., 2007; Lee et al., 2015; Liu et al., 2016). Depending on the type and application of conventional nanocarriers, there are a few methods for transforming them to smart nanocarriers (Bhatia, 2016; Sur et al., 2019; Sirisha, 2020). First, nanocarriers must overcome a number of biological obstacles, including cleaning, making their way to the desired targeted site through the reticuloendothelial system (RES). The RES quickly removes the nanocarrier from circulation and it is then stored in the liver, spleen, or bone marrow as anti-cancer drug payload carrying nanocarriers (Nie, 2010). Second, nanocarriers may be functionalized to distinguish cancer cells from normal cells with pinpoint accuracy. Some proteins are overexpressed on surface-level of cancer cells (Kubler and Albrecht, 2018; Antignani et al., 2020). The smart primary targets are overexpressed proteins (Perez and Fernandez-Medarde, 2015). Nanocarriers are equipped with ligands that match the overexpressed proteins. Smart nanocarriers use ligands to detect cancer cells that have overly expressed receptor proteins of their surface (Sabir et al., 2021). Third, delivering the drug to the target cancerous cells does not imply that the operation is finished. The next major issue will be releasing the drug from the smart carrier while it is being stimulated. The surface of nanocarriers can be grafted with a variety of chemical groups to make them sensitive to the stimuli system (Alvarez-Lorenzo and Concheiro, 2014). Fourth, changes are made to allow anti-cancer drugs to be delivered when combined with another material such as genetic materials (Xu et al., 2014), imaging agents (Das et al., 2020), or even more anti-cancer therapies (Bose et al., 2018).

Smart NPs materials utilized in SDDSs can be classified into Organic and inorganic NPs based on number of organic and inorganic materials have been used to fabricate them with their own distinctive architecture and attached functionalities, and they have been evaluated for effective drug delivery to tumors (Srinivasan et al., 2015). Liposomes, dendrimers, micelles, are example of organic nanoparticles and carbon nanotubes, meso-porous silica NPs (MSNs), gold/silver NPs and Quantum Dots are example of inorganic nanoparticles.

### 5.1 Organic nanocarriers based SDDS

#### 5.1.1 Liposomes

Liposomes are tiny, artificially created vesicles that are completely enclosed by phospholipid bilayer membranes of varying sizes (20–10,000 nm) (Prasanna et al., 2018). Gregoriadis et al. were the first to employ liposomes as an example drug delivery device in 1971 (Gregoriadis et al., 1971). The large unilamellar liposomes (LUV) may then be produced by extrusion of multilamellar vesicles *via* polycarbonate filters, thanks to the invention of novel preparation technique (Prasanna et al., 2018). Liposomes have been widely used as advanced DDSs in numerous clinical trials, especially when the diameter of the liposome was reduced to less than 100 nm (Torchilin, 2012; Akbarzadeh et al., 2013).

The physico-chemical nature of lipids allows drug molecules to be encapsulated or intercalated into phospholipid bilayers, extending the medication’s location. Liposomes have been extensively studied for the delivery of imaging and therapeutic agents in a sustained and controlled manner for cancer diagnosis and treatment, with high diagnostic and therapeutic efficiency and minimal side effects (Koren et al., 2012).

Traditional liposomes have a number of flaws, including instability, insufficient drug loading, faster drug release, and shorter blood circulation times; therefore, they are not smart (Bozzuto and Molinari, 2015). The conventional liposomes need to be Traditional liposomes have been functionalized, making them ideal for use as SDDSs in order to makes them smart for utilized as SDDSs. Liposomes, like other nanocarriers, must overcome the challenge posed by the RES. Liposomes are helped to escape the RES by PEGylation. PEGylated liposomes have a longer blood circulation time as a result (Allen and Cullis, 2013). Smart nanocarriers can distinguish between cancerous and healthy cells. To actively target the cancer site, monoclonal antibodies, antibody fragments, proteins, peptides, vitamins, carbohydrates, and glycoproteins are usually attached/conjugated on the liposome (Sapra and Allen, 2003; Ruoslahti, 2012; Sawant and Torchilin, 2012; Noble et al., 2014). Smart liposomes drug delivery systems are responsive to various external and internal stimulation, including pH change, enzyme transformation, redox reaction, light, ultrasound and microwaves (Jin et al., 2016; Lee and Thompson, 2017). As a smart drug carrier system, ThermoDox, temperature-sensitive DOX liposomes developed by the company Celsion may be the closest formulation to the clinic so far. The doxorubicin may be liberated from ThermoDox at 41.5°C by taking advantage of the dipalmitoylphosphatidylcholine (DPPC) lipid crystallisation melting point (Chen et al., 2014). A novel range of cationic liposome-based systems has also been developed by integrating different cationic lipids for targeted delivery of anionic therapies such as small interfering RNA (siRNA), antisense oligonucleotides, and aptamers etc (Yingchoncharoen et al., 2016). For example, Peddada et al. created a complex nanocarrier by combining a cationic DOTAP (1,2-dioleoyl-3-trimethylammonium-propane) liposome, an anionic copolymer, and an antisense oligonucleotide with a poly (propyl-acrylic acid) (PPAA) polymer backbone. In human ovarian cancer A2780 cells, this complex nanocarrier with grafted poly (alkylene oxides) (g-PAO) increased antisense gene silencing activity. The authors also observed increased antisense oligonucleotide delivery in ovarian tumour xenografts, demonstrating that the DOTAP/PPAA-g-PAO nanocarrier system can be used for antisense oligonucleotide delivery for gene silencing (Peddada et al., 2014). Kang et al. created a dual-targeted liposomal system that used the Pep-1 peptide as a cell penetrating peptide and folic acid as an affinity ligand for the folate receptor (FR). The authors created this dual ligand (Pep-1 and folate)-modified liposome by using a short (PEG-2000) and long (PEG-3400) polymer linker to attach both ligands to the liposomal surface. In FR-positive HeLa and FR-negative HaCaT cells, cellular uptake of various fluorescent tagged liposomes was investigated. In FR positive cells, cellular uptake was higher than in FR negative cells, indicating that this multifunctional liposomal system is suitable for FR-selective drug targeting (Kang et al., 2015).

#### 5.1.2 Micelles

Polymer micelles are thermodynamically stable colloidal solutions formed by self-assembly of amphiphilic block copolymers (O'Reilly et al., 2006). Polymeric micelles are created using block copolymers, which are composed of two or more polymer chains with distinct hydrophilic characteristics. In an aqueous environment, these copolymers spontaneously combine into a core-shell structure. The core is made up of hydrophobic blocks, which may carry any hydrophobic medication, while the shell of hydrophilic blocks (Hibino et al., 2021). To develop therapeutic carriers, a variety of polymeric molecules have been investigated. Polymer-protein conjugates, drug-polymer conjugates, and supramolecular drug delivery systems are just a few examples. Only a few polymers have been accepted into clinical practise out of the many that have been proposed (Sanchez-Moreno et al., 2018). Biodegradable polymers, in particular, are highly preferred due to their high bioavailability, better encapsulation, controlled release, and low toxicity. Wang et al. demonstrated that paclitaxel-loaded micelles bound specifically to an MCF-7 cell-specific phage and found the cytotoxicity of the targeted paclitaxel-loaded phage micelles was significantly higher than that of the free drug or non-targeted micelle formulations against target MCF-7 cells, but not against non-target C166 cells (Wang et al., 2010). Ke et al. created micelles containing both thioridazine (which has been proven to kill cancer stem cells) and doxorubicin, presenting a promising method for breast cancer treatment that targets cancer as well as the cancer stem cells (Ke et al., 2014). Site-specific drug delivery smart nanocarriers are sought in the field of cancer therapy, with different molecules located in the external part of the nanoparticles that favour receptor-mediated cell-internalization (Wang et al., 2014). Different types of ligands, for example, folic acid and peptides, carbohydrates, antibodies, aptamers are utilised to adorn the micelle surface in order to aggressively target cancer cells (Sutton et al., 2007). The core of the micelle can be functionalized to release the anti-cancer medication at the correct concentration. pH gradients, temperature fluctuations, ultrasound, enzymes, and oxidation are among stimuli utilised in micelle based SDDSs (Sutton et al., 2007; Hossen et al., 2019). Co-delivery strategies in cancer treatment are very important for synergetic effects using multifunctional micelles. Seo et al. described a temperature-responsive micelle-based co-delivery system capable of carrying genes and anti-cancer drugs (Seo et al., 2015).

#### 5.1.3 Dendrimers

Dendrimers are synthetic polymers with a high degree of branching made composed of an initiator core and several layers of active terminal groups. Each layer is referred to as a generation (the core is referred to as generation zero), and it is made up of repeating units (Fischer et al., 2010). Dendrimers are great candidates for developing smart nanocarriers for biological applications due to their distinct chemical structure and ability to incorporate a large number of functional groups at spatially precise locations (Nanjwade et al., 2009). Dendrimers are versatile due to their branched structure. Furthermore, all of the surface’s active groups face outward, resulting in a higher drug encapsulation rate. Several kinds of dendrimers have been reported, including poly (propylene-imine) (PPI or POPAM), polylysine dendrimer, dendritic hydrocarbon, carbon oxygen-based dendrimer, porphyrin-based dendrimer, ionic dendrimer, silicon-based dendrimers, phosphorus-based dendrimer, and Newkome dendrimer (Hossen et al., 2019).

Traditional dendrimers are cleared rapidly by the immune system and have a low uptake by cancer cells. The alternative to these limitations is to modify the dendrimer. Chemical modification, copolymerization with a linear polymer, and hybridization with other smart nanocarriers have all been suggested as ways to get around these limitations (Bugno et al., 2015). Peptides, proteins, carbohydrates, aptamers, antibodies, and other substances can be used to modify the surface of dendritic structures to actively target the cancer site (Sirisha, 2020). The surface of the dendrimer may also be changed to respond to various stimuli, such as light, heat, and pH shift protein, and enzyme transformation (Rajasekhar Reddy et al., 2012; Wang et al., 2016). The cationic character of PAMAM, among other dendrimers, makes it ideal for the transport of genetic elements. The production of PAMAM has an impact on delivery efficiency. PAMAM-based nucleic acid delivery was initially reported by Haensler and Szoka in 1993 (Madaan et al., 2014). The use of a dendritic contrast agent for tumour imaging has shown to be highly effective (Hossen et al., 2019). Researchers Zhang and Shi found a multifunctional system that may be used to target cancer treatment using G5-PAMAM dendrimers coated with folic acid and doxorubicin (Zhang et al., 2018). Kaminskas et al. investigated the use of a PEGylated polylysine dendrimer conjugated to doxorubicin to promote controlled and prolonged doxorubicin exposure of lung-resident cancers. After 2 weeks of treatment, they found a 95% reduction in lung tumour burden in rats (Kaminskas et al., 2014).

#### 5.1.4 Polymer based

Smart polymers are extremely efficient polymers that adapt to their surroundings. Natural, semi-synthetic, or synthetic polymers are used to make polymeric NPs (Brighenti et al., 2020). Polymeric nanosystems are formed by the polymerization of numerous monomer units, and under specific conditions, they may be structured and self-assemble with a nanometric size (10–100 nm) (Joglekar and Trewyn, 2013). Drugs can be entrapped, encapsulated, or bonded to polymeric NPs in the form of a nanosphere, a nano-capsule, or a drug conjugate, depending on the production technique (Prabhu et al., 2015). Polymeric capsules may be created by conjugating targeting ligands, which boost selectivity for cancer cells and improve intracellular drug delivery while decreasing various side effects and medication toxicity (Prabhu et al., 2015). Monoclonal antibodies (mAbs) or antibody fragments, aptamers, peptides, and small compounds, such as folic acid, are widely used as targeting ligands for polymeric capsule (Avramovic et al., 2020). These ligands specifically bind to antigens or receptors overexpressed on cancer cells (Rana and Bhatnagar, 2021). The efficacy of polymeric carriers modified with targeting ligands is determined by ligand properties such as density and receptor binding affinities, which can improve receptor internalisation and drug biodistribution. A drug is chemically bonded to the polymer *via* a linker/spacer in drug-conjugates. When the drug is released at the target site, the bond drug-linker/spacer is a common breakage point. FA-PEG-b-PCL-hyd-DOX, a multifunctional polymeric-drug conjugate containing a di-block PEG-PCL copolymer linked to DOX through a labile hydrazone bond and adorned with folic acid (FA), was developed by Guo et al. (Guo et al., 2016)]. Hu et al. created a nanoplatform with paclitaxel (PTX) encapsulated in a triblock PCL-PEG-PCL copolymer that confirmed sustained drug release and a lower cytotoxic effect when compared to free PTX injection (Hu et al., 2017). Guo et al. demonstrated the ability of the hydrophobic polymer PLGA to encapsulate the low-solubility medicine PTX in a poly (lactic-co-glycolic acid)-poly (ethylene glycol) (PLGA-PEG) nanoplatform, with longer circulation time and improved cancer inhibition confirmed when this SDDS was decorated with DNA aptamers in C6 glioma cells (Guo et al., 2011). In another study, Wang et al. found that methoxy PEG-PLGA NP co-loaded with hydrophilic DOX and hydrophobic PCT inhibited cancer development more effectively than polymeric micelles loaded with only one medication (either DOX or PCT), with the best anticancer effectiveness at a 2:1 concentration ratio (Yingchoncharoen et al., 2016). Duong et al. also developed a PEG-PLGA copolymer system for the delivery of DOX and PCT, which includes the targeting ligand folate and the TAT peptide, and which improves the cellular interaction between PEG-PLGA micelles in the kB cell line of a human oral cavity carcinoma (Duong, 2013). In essence, folate improves the drug carriers’ targeting ability, whereas TAT peptide is a cell-penetrating peptide (CPP) used to modify the carrier surface. In PEG-PLGA micelles, different concentration ratios of DOX and PCT were used, and a concentration ratio of 1:0.2 was found to be more effective than a concentration ratio of 1:1 (Duong, 2013). Jin et al. recently developed a promising smart delivery system based on the cationic deblock poly (ethyleneimine)-poly (lactic acid) (PEI-PLA) copolymer, which was designed to deliver the drug PTX and siRNA in a synergistic strategy in chemo or gene therapy for non-small cell lung cancer (Jin et al., 2018). This PTX NPs formulation enhances the drug’s effect by inhibiting target proteins involved in cancer cell metabolism and proliferation *via* siRNA. With high drug loading, a longer half-life in the circulation, lower toxicity, and an antiproliferative effect of PTX on A549 cells, this co-delivery system is a promising SDDS (Jin et al., 2018).

### 5.2 Inorganic based SDDSs

#### 5.2.1 Carbon nanotubes (CNTs)

CNTs have attracted incredible interest in the biomedical field due both to their promising properties (such as high surface area, needle-like structure, considerable strength, flexible interaction with drug cargo, high drug loading capacity, outstanding optical and electrical features, high stability, biocompatibility, and ability to release therapeutic agents at targeted sites) and negative properties (most notably, lack of biodegradability and toxicity) (Alshehri et al., 2016; Costa et al., 2016; Singh et al., 2016; Azqhandi et al., 2017). CNTs are one-dimensional carbon allotropes with a nanostructure with a length-to-diameter ratio greater than one million that are made by rolling a thick sheet of graphene into a smooth cylinder with a diameter on the order of a nanometre (nm) (Rahamathulla et al., 2021). CNT can be fabricated in a number of ways, including rolling up a single layer of graphene sheet (single-walled CNT; SWCNT) or rolling up many layers to form concentric cylinders (multiwalled CNT; MWNT) (Rahamathulla et al., 2021). Traditional CNTs have difficulties dissolving in both aqueous and organic solvents, which makes it difficult to disperse homogeneously as compared with other nanoparticles. To make conventional CNTs smart, they must be functionalized chemically or physically (Li Z. et al., 2017). Several biological applications, including as proteins, nucleic acids, and drug transporters, have been successfully explored using CNTs that have been functional (Anzar et al., 2020). PEGylation is a critical step in increasing solubility, avoiding RES, and reducing toxicity (Kenchegowda et al., 2021). The polymer poly (N-isopropyl acrylamide) (PNIPAM) is temperature sensitive. PNIPAM could be used to modify CNTs for temperature stimulus because of their low critical stimulus temperature (LCST) (Schmaljohann, 2006). For enzyme-responsive drug release, a disulfide cross-linker based on methacrylate cysteine is used. An ionizable polymer with a pKa value of 3–10 is an ideal candidate for pH responsiveness. Weak acids and bases show a change in the ionization state upon pH variation (Schmaljohann, 2006). Researchers developed a PEGylated CNT complex loaded with paclitaxel for the treatment of breast cancer in an early study. When compared to free paclitaxel alone, the CNT-paclitaxel complex showed better treatment efficacy in a 4T1 murine breast cancer model (Liu et al., 2008). Jain et al. reported that chemical modification of CNTs by carbohydrate d-galactose can generate a novel cascade of chemical functionalization of MWCNTs (Jain et al., 2009). Galactosylated MWCNTs are utilised to deliver active ligands (like galactose) to tumour sites as a targeted drug (Jain et al., 2009). SWCNTs are more efficient in drug distribution than MWCNTs because their walls are more defined and MWCNTs have more structural flaws. CNTs have been studied as nanocarriers for medication delivery as well as biomolecules including DNA, siRNA, and others. Functionalized carbon nanotubes can be utilised as early cancer detection techniques (Hossen et al., 2019). Cheng et al. recently developed a PLGA-functionalized CNT system for delivering the proapoptotic protein caspase-3 (CP3) to bone cancer cells with reduced toxicity (Cheng Q. et al., 2013). This nanocomplex showed efficient transfection of CP3 in cells and suppressed their proliferation. In a CNT-PLGA system, transcription factors were well delivered with a good transfection rate, and the payload release profile could be modified by adjusting the PLGA polymer molecular weight and ratio (Cheng Q. et al., 2013). For the treatment of cancer, Mehra et al. created a multiwall PEG-CTN complex loaded with doxorubicin (DOX) (Mehra and NKJain, 2015). On the surface of this DOX/ES-PEG-MWCNT system, both folic acid (FA) and estrone (ES) were attached as targeting molecules. They observed a long survival of Balb/c mice with MCF-7 tumors treated with DOX/ES-PEGMWCNT nano formulation (Mehra and NKJain, 2015).

#### 5.2.2 Meso-porous silica nanoparticles (MSNs)

Mesoporous materials, which have pore sizes ranging from 2 to 50 nm, high surface areas, adjustable pore sizes and internal architectures, and a plethora of modifiable sites, are frequently utilised in catalyzer, sensor, and molecular sieve research (Shi et al., 2020). In the recent decades, mesoporous materials have shown significant potential for SDDS. Due to their drug loading capacity, desirable biocompatibility, and practical feasibility, MSNs have attracted the attention of researchers (Farjadian et al., 2019). The versatility of MSNs is due to their tuneable particle size (50–300 nm), tuneable pore size (2–6 nm), high surface area, and biocompatibility (Hossen et al., 2019). Tuneable particle size is an essential criterion to be a smart nanocarrier, and tuneable pore size allows drugs of different molecular shapes to be loaded. The high surface areas of the pores and external surface are suitable for grafting different functional groups on MSNs (Yingchoncharoen et al., 2016; Hossen et al., 2019). Typical MSNs have low circulation half-lives due to hemolysis of red blood cells, non-specific binding to human serum proteins, and phagocytosis of human THP-1 mono-cytic leukemia macrophages. PEGylation can help to reduce the negative impact of these variables (He et al., 2010). MSNs are used as stimulus-sensitive drug delivery systems, and the surface pores are also blocked to build gatekeeper-based delivery systems, thanks to their adaptability. For targeted administration of doxorubicin, Cheng et al. developed and synthesised a pH responsive multifunctional MSN system made up of poly dopamine, poly (ethylene glycol), and folic acid (Cheng et al., 2017). The findings revealed significant anticancer activity and release of the encapsulated drug payload from MSNPDA-PEG-FA nanosystems in acidic pH (Cheng et al., 2017). Yang et al. created disulfide-bridged ‘degradable dendritic mesoporous organo-silica nanoparticles (DDMONs) to deliver therapeutic proteins to cancer cells (Yang et al., 2016). In B16F0 cancer cells, this DDMONs system demonstrated a greater rate of glutathione (GSH)-responsive degradation and release of the therapeutic protein, but in normal HEK293t cells, the nanoparticle degradation was modest (Yang et al., 2016). Targeted MSNs therapies work by interfering directly with specific molecules involved in cancer growth and progression or indirectly by activating the immune system to detect and destroy cancer cells to prevent cancer from spreading (Colilla et al., 2010). For example, many anticancer medicines require “zero release” before reaching the target site. Efficient distribution of doxorubicin (DOX) utilising MSNs coated with a PEG copolymer employing 50 nm MSNs, which can reach a size of 110 nm when coated with the copolymer (Gary-Bobo et al., 2012; Bharti et al., 2015).

#### 5.2.3 Gold nanoparticles

As GNPs (gold nanoparticles) have a high drug loading capacity, biocompatibility, and stability, they can be used as nano-carriers to transport drugs. In order to create GNPs with the desired morphology, the seeded growth technique is used (Dhanasekaran, 2015). The size of GNPs can be controlled by adjusting the seed to chloroauric salt ratio and the pace at which reducing agents are added, and the form of GNPs may be controlled by using surfactant intelligently to tailor the end facets (Wang et al., 2020). Medicines are connected to the surface of GO in spherical or rod-shaped GNPs; in hollow-structured GNPs, drugs are enclosed in the hollow cave (Shi et al., 2020). Non-covalent and covalent interactions are involved in the conjugation of GNPs and medicines (Wang et al., 2020). Among the non-covalent interactions are electrostatic and hydrophobic interactions, which are weak forces linking drug payload molecules to GNPs (Shi et al., 2020). The gold–thiolate bond (Au–S) is primarily responsible for covalent connections, and thiol-containing molecules are connected on the surface of GNPs in this fashion (Xue et al., 2021). For example, thiol-linked drugs or genes are linked on the surface of GNPs to release drug delivery; thiol-linked targeting groups are also decorated on the surface to improve targeting efficacy; and polymers with stimuli responsibility are functionalized on GNPs *via* Au–S link or electrostatic attraction, endowing the system with TME responsibility (Cobley et al., 2011). The surface plasmon resonance (SPR) phenomena in GNPs is particularly fascinating, which allows them to change light into heat and disperse that heat to kill cancer cells (Sztandera et al., 2019). Ideally, SDDS should be chemically stable in biological media, biocompatible, and targetable. Traditional GNPs are unstable in blood and are more likely to be absorbed by RES. In order to overcome these limitations, gold nanocarriers must be PEGylated. PEGylated GNPs exhibit enhanced solubility and stability under physiological conditions (Qian et al., 2011). GNPs can be modified by ligands or tumor-specific recognition molecules to deliver targeted drugs such as transferrin, folic acid, epidermal growth factor (EGF), or any number of monoclonal antibodies can be conjugated to the surface of GNPs (Vines et al., 2019). Drugs can be released from GNPs through either (1) external stimulation (laser, ultrasound, X-ray, light) or (2) internal stimulation (pH, redox condition, matrix metalloproteinase) (Tian et al., 2016; Yao et al., 2016). Trastuzumab (anti-EGF receptor monoclonal antibodies) was conjugated with citrate-coated GNPs to target EGF receptors in human SK-BR-3 breast cancer cells, resulting in downstream expression of EGF receptors and a 2-fold increase in trastuzumab cytotoxicity, even at low GNP concentrations (Jiang et al., 2008). Another study used GNPs to treat pancreatic cancer with gemcitabine and cetuximab. The cancer site could be identified using GNPs conjugated with fluorescently labelled heparin (Bansal et al., 2020).

#### 5.2.4 Quantum dots (QDs)

QDs are semiconductor nanoparticles with excellent photoluminescence properties, optical properties, and electronic properties that make them suitable for image guided drug delivery. (Mi, 2020). This smart carrier could be used to visualise the tumour and can be functionalized with the targeting ligands for tissue specific therapeutic delivery application for the drug is delivered to the desired location. Various targeted QDs have been studied for diagnosis and therapeutic delivery applications over the years (Badıllı et al., 2020). Chen et al., for example, developed a quantum dot-based FRET system for image-guided drug delivery in the nucleus (Chen H. et al., 2015). In this study, graphene quantum dots (GQDs) were prepared and decorated with TAT peptide to facilitate nuclear localization. The quantum dot-based FRET system enabled real-time monitoring of therapeutic delivery as well as image-based tracking of release (Chen H. et al., 2015). Recent research has shown that conjugating metal based QDs with lipid nanocarriers reduces their cytotoxicity and improves their safety (Olerile et al., 2017). Iannazzo et al. recently demonstrated the potential of graphene QD-based targeted drug delivery. To exploit the biotin receptor overexpressed on tumour cells, they covalently conjugated QDs to the tumour targeting ligand biotin. This system utilizes the pH stimuli to release the drug payload at desired targeted site (Iannazzo et al., 2017). The inherent florescence of QDs makes them ideal for cancer imaging. Ovarian cancer has been diagnosed using a folic acid complex (Zhao, 2016). A DNA aptamer was added up to the top of the created QDs to target mutant MUC1 mucin, which is overexpressed in ovarian cancer. Doxorubicin was attached to the surface of the QD by a pH labile hydrazine linker, which hydrolyses at the acidic pH of the tumour microenvironment, allowing for regulated drug release (Dutta et al., 2021).

### 5.3 Antibody based SDDSs

In the past 3 decades, monoclonal antibodies have evolved from scientific tools into powerful therapeutics. A monoclonal antibody (mAb) is covalently attached to a cytotoxic drug payload *via* a chemical linker in an antibody–drug conjugate (ADC). It combines the advantages of highly specific targeting and a highly potent killing effect to achieve accurate and efficient cancer cell elimination, and it has become one of the hotspots for anticancer drug research and development (Fu et al., 2022). The first ADC drug, Mylotarg^®^ (gemtuzumab ozogamicin), was approved by the US Food and Drug Administration (FDA) in 2000 for adults with acute myeloid leukaemia (AML), signalling the start of the ADC era of cancer targeted therapy (Norsworthy et al., 2018).

**FIGURE 9:**
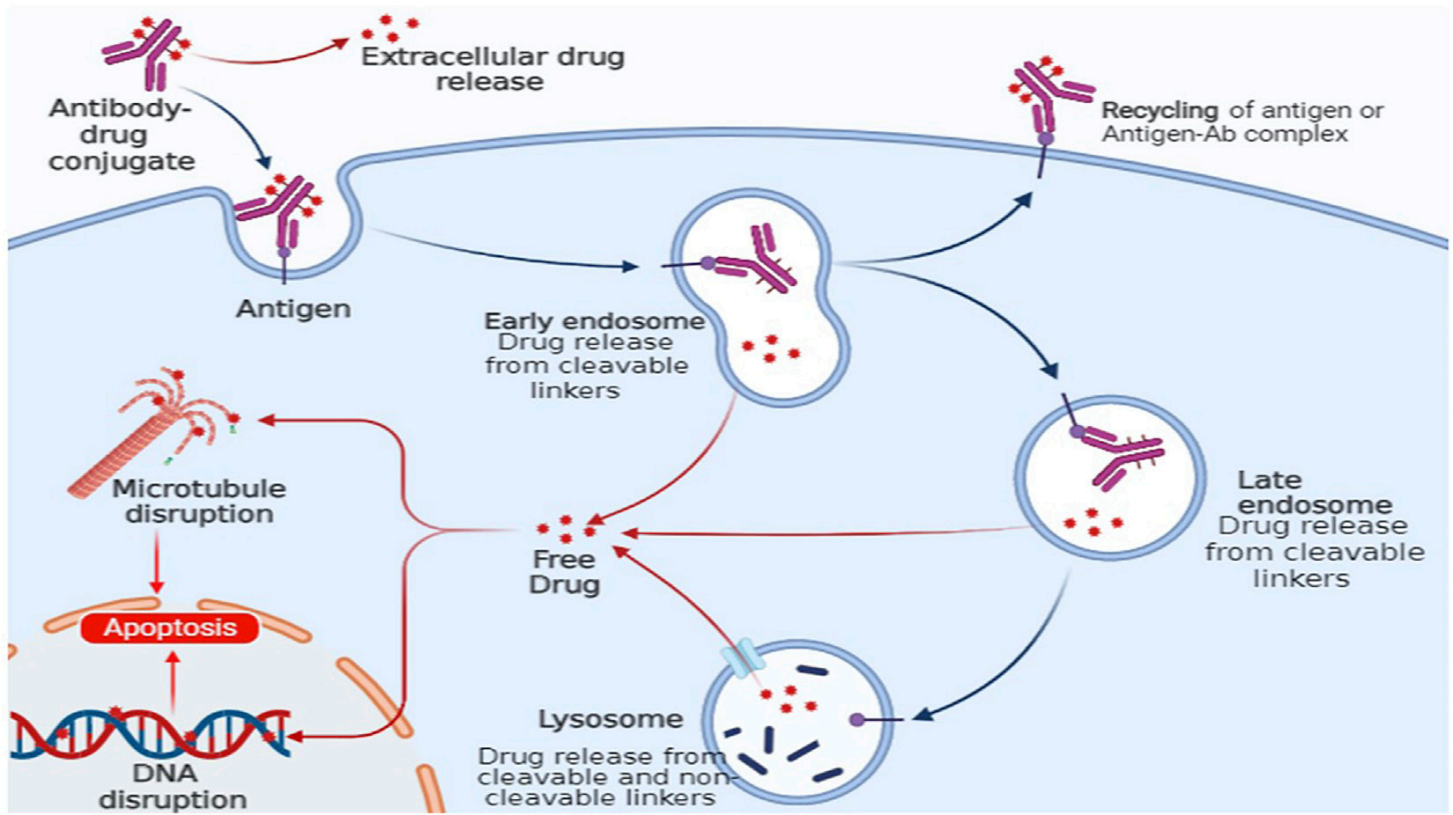
The general mechanism of action of an antibody-drug conjugate (ADC).

By December 2021, 14 ADC drugs had been approved worldwide for both haematological malignancies and solid tumours. Furthermore, there are currently over 100 ADC candidates in various stages of clinical trials (Fu et al., 2022). Figure 9 depicts the general mechanism of action for an ADC. Following administration, the ADC’s mAb component recognises and binds to the target tumour cells’ cell surface antigens. After antigen binding, the ADC–antigen complex is internalised by the cancer cell through endocytosis (Ritchie et al., 2013). In the case of non-cleavable linkers, the internalised complex is broken down *via* proteolysis within lysosomes, releasing the cytotoxic payload inside the cell, whereas the mechanism of payload release for ADCs with cleavable linkers varies depending on the specific linker used (Sanadgol and Wackerlig, 2020). The liberated payload binds to its target in all cases, causing cell death through apoptosis (Tong et al., 2021).

### 5.4 Small molecule drug conjugate based SDDSs

Just like ADCs, Small Molecule Drug Conjugates (SMDCs) are another class of SDDSs. An SMDC comprises of a targeting ligand, a releasable bond, a hydrophilic spacer, and a therapeutic drug payload (Rana and Bhatnagar, 2021). SMDCs which use biomarker-targeted small molecule compounds as the targeting moieties, in contrast to ADCs, provide a new, less established approach to targeted delivery. However, SMDCs have several advantages, including 1) a non-immunogenic nature, 2) a much more manageable synthesis, and 3) lower molecular weights, all of which contribute to a high potential for cell penetration in solid tumours (Zhuang et al., 2019). SMDCs have been successfully targeted against Folate Receptor, Prostate Specific Membrane Antigen, Somatostatin Receptor, and Carbonic Anhydrase IX (Ghiasikhou et al., 2019). More recently, other receptors (such as biotin receptor, bombesin receptor, Eph receptor) have gained attention as they can be potentially targeted with small molecules (Rana and Bhatnagar, 2021). Among them, the folate receptor (Iannazzo et al.) has received most of the attention and several compounds primarily developed by Endocyte have entered clinical trials (Zhuang et al., 2019). The first compounds that used folate as a targeting moiety were used for imaging. Etarfolatide was one of the first products in its class to make it to the clinic (Patel et al., 2021). A SMDC most commonly used as a delivery vehicle for folic acid is vintafolide, a conjugate containing desacetylvinblastin hydrazide (DAVLBH). Developed at Endocyte and later licensed to Merck in a $1 billion deal, this drug reached phase III clinical trials for platinum-resistant ovarian cancer. However, the results of the clinical trials, reported shortly after the European Medicines Agency (EMA) had recommended the drug approval, halted its development (Vlahov et al., 2017; Reddy et al., 2018). An innovative compound in the clinic, the peptide based SMDC 177Lu-DOTATATE, has been approved by the USFDA and EMA for the treatment of gastroenteropancreatic neuroendocrine tumors (Curtis et al., 2014). Several recent preclinical studies demonstrated the striking potency of various chemotherapeutic agents such as PEN-866, EC145, AEZS-108, NGR-TNF (Asn-Gly-Arg-TNFa), and EC0225 in xenograft models of solid tumours including breast, pancreatic, and small cell lung cancer in xenograft models of solid tumours including breast, pancreatic, and small cell lung cancer (SCLC) (Patel et al., 2021). In theory, SMDCs can deliver cytotoxic agents to target cells that overexpress specific receptors such as FR, PSMA, and others by targeting ligand to the receptors and allowing it to be internalised *via* receptor-mediated endocytosis (Leamon and Jackman, 2008). Once the SMDC–receptor complex is internalised, it travels from the endosome to the lysosome, where the cytotoxic drug is released from the SMDCs *via* deconjugation (cleaving the linker) in intracellular compartments, resulting in cell death (Patel et al., 2021). SMDCs targeting cancer endocytosis, heat shock protein 90 (HSP90), BCR/ABL fusion protein, PSMA, GLUT1, LRP1, aminopeptidase N (APN), and somatostatin receptor are all in clinical trials (SSTR). All of them are currently undergoing clinical trials in various stages (Table 2). The SMDC approach, on the other hand, has been widely used in the fields of radiotherapy and cancer diagnosis. The efficacy of ligand-targeted compounds used for cancer imaging has been demonstrated in clinical trials by the identification and localization of tumours (Srinivasarao et al., 2015; Srinivasarao and Low, 2017; Banerji et al., 2018; Patel et al., 2021; Rana and Bhatnagar, 2021).

**TABLE 2:** Some medicines for cancer treatment based on SDDSs that are in clinical trials or already commercialized.

Product	Targeting	Smart Carrier	Drug Payload	Stimuli	Indication	Clinical status	Identifier
Doxil	Passive	PEGylated liposome	Doxorubicin	****	Ovarian cancer, AIDS-related Kaposi’s sarcoma, breast cancer, myeloma	Approved 1995 by FDA	****
DaunoXome	Passive	Liposome	Daunorubicin	****	HIV-associated Kaposi’s sarcoma	Approved 1996 by FDA	****
Myocet	Passive	Non-PEGylated liposomal	Doxorubicin	****	Metastatic breast cancer	Approved 2000 by EMEA	****
Lipusu	Passive	Liposome	Paclitaxel	****	Ovarian cancer, non-small cell lung cancer	Approved 2003 by CFDA	****
Nanoxel	Passive	Polymeric micelle	Paclitaxel	****	Breast cancer, non-small-cell lung cancer, and ovarian cancer	Approved 2006 by CDSCO	****
Marqibo	Passive	Liposome	Vincristine Sulfate	****	Philadelphia chromosome-negative acute lymphoblastic leukemia	Approved 2012 by FDA	****
Kadcyla (ado-trastuzumab Emtansine)	Active	Anti-HER2 tumor cell specific antigen	Maytansinoid DM1	pH	Early Breast Cancer	Approved 2013 by FDA	****
Gemtuzumab ozogamicin	Active	Anti-CD33 tumor cell specific antigen	Calicheamicin	pH	Acute myeloid lymphoma	Approved 2017 by FDA	****
Besponsa (Inotuzumab ozogamicin)	Active	Anti-CD22 tumor cell specific antigen	Calicheamicin	pH	Relapsed acute lymphoblastic Leukemia	Approved 2017 by FDA	
Brentuximab vedotin	Active	Anti-CD30 tumor cell specific antigen	Monomethyl Auristatin E (MMAE)	Enzyme	Relapsed Hodgkin’s lymphoma and anaplastic large cell lymphoma	Approved 2022 by FDA	****
Depatuxizumab mafodotin	Active	Anti-EGFR tumor cell specific antigen	Monomethyl Auristatin E (MMAE)	****	Glioblastoma	Phase III	NCT02573324
Enfortumab vedotin	Active	Anti-EGFR tumor cell specific antigen	Monomethyl Auristatin E (MMAE)	Enzyme	Advanced urothelial cancer	Phase III	NCT04136808
Vintafolide (EC145)	Active	Folic acid	Desacetylvinblastine	pH	Solid tumors,	Phase I (completed)	NCT01002924
					Recurrent or refractory solid tumors,	Phase II (completed)	NCT00308269
					Platinum resistant ovarian cancer	Phase II (completed)	NCT00722592
					FR (++) second line non-small cell lung cancer	Phase II (completed)	NCT01577654
Vintafolide (EC145) + Etrafolide (EC 20)	Active	Folic acid	Desacetylvinblastine	pH	Ovarian cancer, endometrial cancer	Phase II (completed)	NCT00507741
					Adenocarcinoma of lungs	Phase II (completed)	NCT00511485
EC1456 and EC20	Active	Folic acid	Tubulysin	pH	Solid tumors, non-small cell lung carcinoma	Phase I (completed)	NCT01999738
Glufosfamide	Active	Glucose	Fluorouracil	****	Second line metastatic pancreatic cancer	Phase III (recruiting)	NCT01954992
MAGNABLATE I	Passive	Iron oxide magnetite	Doxorubicin	Magnetic	Prostate cancer	Phase I	NCT02033447
NC6300	Passive	Polymeric micelles	Epirubicin	pH	Solid tumor, soft tissue sarcoma, metastatic sarcoma, sarcoma	Phase I and II	NCT03168061
(MTC-DOX)	Passive	Iron and carbon	Doxorubicin	Magnetic	Unresectable hepatocellular carcinoma	Phase II and III	NCT00034333
					Hepatocellular carcinoma	Phase I and II	NCT00054951
					Liver metastasis	Phase I and II	NCT00041808
ThermoDox	Passive	Liposome	Doxorubicin	Temperature	Recurrent regional breast cancer	Phase I and II	NCT00826085
					Liver tumor	Phase I	NCT02181075
					Pediatric refractory solid tumor	Phase I	NCT02536183
			Doxorubicin combined with high Intensity focused ultrasound (HIFU)	Temperature	Painful bone metastasis, breast carcinoma, non-small cell lung cancer, small cell lung cancer, adenocarcinoma	Phase II	NCT01640847

### 5.5 Aptamers based SDDSs

An aptamer is a simple, small, single-stranded deoxyribonucleic acid (DNA) or ribonucleic acid (RNA) that folds into a three-dimensional conformation just like an antibody for binding to target molecules (Nimjee et al., 2017). Aptamers can typically bind to various molecules, such as overexpressed receptors, for diagnostic and therapeutic purposes using an *in vitro* iterative selection method known as SELEX (Systematic Evolution of Ligands by Exponential Enrichment) (Maghsoudi et al., 2019). Aptamers are more beneficial, less toxic, and easier to modify and synthesise in the lab than antibodies. Furthermore, aptamers were chosen as a new family of cancer therapeutics because of their numerous advantages over recent cancer therapies such as monoclonal antibodies. Their promising affinity for specific tumour cell lines, higher robustness than antibodies, fast *in vitro* selection, low immunogenicity, and better penetration into solid tumour tissue are just a few of these advantages (Hori et al., 2018). Antisoma developed AS1411, a 26-nt guanosine-rich G-quadruplex DNA oligonucleotide that was the first aptamer to enter clinical trials for cancer treatment. AS1411 was discovered in a screen for antiproliferative DNA oligonucleotides, not by SELEX (Hori et al., 2018). Aptamer–drug conjugates are particularly useful in the treatment of chemotherapeutic agents with systemic side effects. Doxorubicin (Dox) has been used as a model agent for cell-specific aptamer conjugation. Dox is a traditional chemotherapeutic agent that induces cancer cell death by intercalating into DNA. Dox can non-covalently conjugate to aptamers *via* intercalation into their GC-rich regions for delivery into specific cells, according to some studies (Bagalkot et al., 2006; Hu et al., 2012; Subramanian et al., 2012). Several other groups have reported novel types of aptamer–Dox conjugates in the recent years. Wen et al. isolated a CD38-targeting DNA aptamer and used CG-cargo to non-covalently conjugate Dox to it in a CG-repeat structure (Wen et al., 2016). The aptamer-Dox conjugate was formed with a 1:5 M ratio of aptamer to Dox using the CG-repeat structure. It specifically released Dox in tumour cells when systemically administered to multiple myeloma-bearing mice, inhibiting tumour growth and improving mouse survival rates (Wen et al., 2016). Trinh et al. developed AS1411-Dox, a drug-DNA adduct, by crosslinking Dox and AS1411 with formaldehyde overnight at 10°. AS1411-Dox inhibited tumour growth in hepatocellular carcinoma-bearing mice without causing severe toxicity in non-tumor tissues when given systemically (Trinh et al., 2015). Covalent conjugation to aptamers has also been utilized to target other chemotherapy agents to cancer cells. For example, Zhao et al. developed a cell-specific aptamer—methotrexate (MTX) conjugate to specifically inhibit AML (Zhao et al., 2015). In the first step, they isolated a DNA aptamer that targets CD117, an antigen that is highly expressed on AML cells. MTX was covalently conjugated with DNA aptamers with G-quadruplex structures using N-hydroxysuccinimide (NHS). The CD117 aptamer-MTX conjugate specifically inhibited the cell growth in AML (Zhao et al., 2015).

## 6 challenges and the future perspectives

It is inevitable that every opportunity will come with some challenges. There is no exception to this rule with SDDSs. In order for SDDSs to succeed, they must overcome the toxicity of nanocarriers in the human body, the cost-effectiveness of the system, the heterogeneity and diversity of cancers, and the lack of specific regulatory guidelines (Shi et al., 2017).

To kill cancer cells, smart carriers needs to transport and release anti-cancer drugs at the targeted sites. In nanocarrier delivery, the biggest challenge is the toxicity of nanocarriers, which will need to be studied further in the future, as well as the limitation between their use in small animals and their clinical effectiveness. Depending on the chemical composition, size, shape, specific surface area, surface charge, as well as the presence or absence of a shell around the nanocarrier, conventional nanocarriers can accumulate in different vital organs including the lungs, spleen, kidneys, liver, and heart. Similarly, in case of ADCs, the high cost of production, limited penetration into solid tumor masses, and premature drug release the main concerns (Lo et al., 2022).

The challenges associated with ligands include selection of an appropriate ligand, developing conjugation strategies, and characterizing the release of the drug from the ligand (selection of a linker). A carrier-based challenge involves carrier selection and carrier physicochemical and pharmacokinetic characterization. SDDSs formulation requires additional steps in chemical synthesis and purification. Furthermore, there are additional quality control and regulatory steps, increased costs, and longer timeframes. The majority of these carriers have been designed and tested in small animal models, with excellent therapeutic results; however, the translation of animal results into human success has been limited. In order to fully comprehend the advantages and disadvantages of these vehicles, more clinical data is needed.

Another challenge that limits application of SDDSs is functional group complementarity as well as release of drug in active form in cellular melieu. pH-responsive delivery can be accomplished by the controlled protonation of the functional groups in the linker of SDDS and pH-responsive bond cleavage. Similarly, pH-induced bond cleavage can release drugs directly or by breaking up the carrier’s topological structure. Chemical bonds that can be cleaved by pH-responsive materials include hydrazine, oxime, amide, imine, ketal or acetal, orthoester, and phenyl vinyl ether. When drugs are linked to the carrier by these bonds, their cleavage in an acidic environment leads to their release. For the redox-responsive system, commonly used linkers include disulfide and diselenide linkages which will be broken with significant increases in the level of surrounding reducing agents such as GSH. On similar lines, it is necessary for enzyme-responsive SDDSs to tolerate specific conditions of pH and presence of other ions in cellular mileiu that may interfere with enzyme activity. Besides in the DDS the substrate should mimic and also be complementary to the binding pocket of target enzyme for the targeted enzyme to act, the actions of the targeted enzyme must alter the properties of the linkers used in SDDS as well. It is also worth mentioning that the long-term effects of associated with toxicity due to accumulation of nanocarriers or other SDDDs in patients is a necessity that requires investigation. All the above are formidable challenges for medicinal chemists; however, the potential of SDDSs in translational medicine cannot be denied.

In the future, SDDS will combine diagnosis and targeted therapy into one, centralized treatment system. A novel theranostic strategy has the potential to facilitate highly selective, effective, and relatively sensitive treatments of cancer and other chronic diseases, leading to personalized chemotherapy with improved outcomes for patients. All smart drug delivery systems all share the same goal: to benefit patients. Future research on smart DDSs for controlled drug delivery should concentrate on clinical translation so that more stimulus-sensitive nanomedicine may be employed in clinical settings.

## 7 Conclusion

The pharmaceutical and biotechnology industries are undergoing a significant transformation. Although the last decade has seen significant advances in drug delivery yet challenges remain. Smart drug delivery systems have the potential to overcome the limitations of traditional drug delivery methods. The development of smart drug delivery systems holds a lot of promise for pathology-specific medication design and delivery techniques that are tailored as per therapeutic needs.

Smart drug delivery systems incorporate several benefits, which includes i) a long shelf life and is not readily degraded or cleared by the reticuloendothelial system (RES) during blood circulation, ii) efficient intracellular drug delivery at the tumour targeted region or location that meets iii) drug pharmacodynamics of kinetics and spatial control, and iv) tolerability.

Another class of SDDSs include stimuli-responsive nanocarriers that can be used to deliver diagnostic and therapeutic substances to specific locations. Many improvements in stimulus sensitive delivery systems have been made in the last few years. In this review, literature studies of internal stimuli such as pH, redox, and enzyme demonstrate a superior property of controlling and adjusting the location and time of drug release without the use of any other external remote apparatus, leading in increased therapeutic drug internalisation in target cells and external stimuli such as light, ultrasound, and magnetic fields can also be utilised to initiate or increase drug release at disease sites. Smart Nanocarriers, a marvel of modern technology, are critical in the delivery of anti-cancer drugs. Because of their outstanding characteristics for cancer therapy, organic and inorganic based smart nanomaterials have recovered a lot of interest.

Changes will be made in clinical trials to allow for the specific targeting of cancer cells, which will enhance cancer patients’ quality of life by minimising the side effects of chemotherapeutic drugs and improving overall survival. Liposomes, nano-suspension, polymer nanoparticles, nanocapsule, micelles, doxil, and other nanocarriers have been authorised in clinical trials (Mi, 2020).

SDDS has a bright future and offers many opportunities for improving quality of life and patient compliance, and it could become the future of Translational Medicine.
